# Near‐Infrared Cocrystal Nanofluorophore with Enhanced Two‐Photon Absorption Cross Sections

**DOI:** 10.1002/advs.202523319

**Published:** 2026-01-15

**Authors:** Liangmeng Hao, Ying Ni, Jiawei Huang, Yucheng Wang, Fan Liu, Xu Wang, Lei Kang, Zheshuai Lin, Weigang Zhu

**Affiliations:** ^1^ State Key Laboratory of Advanced Materials for Intelligent Sensing Key Laboratory of Organic Integrated Circuits Ministry of Education Tianjin Key Laboratory of Molecular Optoelectronic Sciences Department of Chemistry School of Science Tianjin University Tianjin China; ^2^ Songshan Lake Materials Laboratory Dongguan China; ^3^ College of Chemistry Chemical Engineering and Materials Science Key Laboratory of Molecular and Nano Probes Ministry of Education Collaborative Innovation Center of Functionalized Probes for Chemical Imaging in Universities of Shandong Shandong Normal University Jinan China; ^4^ Functional Crystal Laboratory Technical Institute of Physics and Chemistry Chinese Academy of Sciences Beijing China

**Keywords:** cocrystal, imaging, nanoparticle, theory, two‐photon absorption

## Abstract

The growing demand for organic nanoprobes that combine broadband two‐photon absorption (TPA) with near‐infrared (NIR) optical excitation continues to drive advancements in biological imaging and advanced photonics. However, the design and preparation of such nanoprobe materials remain a significant challenge. Here, we employ co‐crystallization strategy to fabricate a TPA crystal material, BP4TC (donor BP4VA, acceptor TCNB), exhibiting deep red fluorescence. Nanoprecipitation with an amphiphilic surfactant yields water‐dispersible BP4TC nanoparticles (BP4TC‐NPs, ≈50 nm), which are subsequently used for open‐aperture Z‐scan measurements. This marks the first experimental determination of the TPA cross‐section for a molecular multicomponent solid. Prominent reverse‐saturable and saturable absorption characteristics span 700–1000 nm, with corresponding TPA cross‐sections decreasing monotonically with increasing wavelength. First‐principles calculations demonstrate that BP4TC exhibits a stronger intermolecular charge transfer capacity, verifying its enhanced TPA ability. The TPA cross‐section of BP4TC was further computed using the TDDFT approach, and the result indicates that BP4TC is a potent two‐photon absorber. Under 900 nm excitation, BP4TC‐NPs produce bright, low‐background fluorescence in A549 cells (human lung cancer cells), exhibiting negligible cytotoxicity. Notably, by combining broadband NIR‐I TPA with favorable biological properties, these cocrystal materials establish a multifunctional platform for biological multiphoton imaging and NIR photonics, while providing an experimentally validated blueprint for advanced nonlinear optical nanomaterials.

## Graphical abstract

In this study, organic cocrystal nanoparticles (BP4TC‐NPs) were prepared via a nanoprecipitation method using an amphiphilic surfactant. The resulting cocrystal nanoparticles exhibit exceptional nonlinear optical properties and bioimaging capabilities. Open‐aperture Z‐scan experiments revealed a broad TPA response spanning the NIR‐I/II regions (700–1000 nm), with a maximum cross‐section of 272 GM at 700 nm. Furthermore, the TPA cross‐section was computed using the Time‐Dependent Density Functional Theory approach. In vitro cell models confirm its strong two‐photon‐excited fluorescence imaging capability. The probe exhibits bright, high‐contrast intracellular emission, and negligible cytotoxicity.

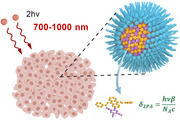

## 1 Introduction

Since the advent of the first laser in 1960, nonlinear optical (NLO) phenomena have stimulated intense exploration. At low intensities a material's optical response is linearly proportional to the electric‐field amplitude; at high intensities, however, light–matter interactions become markedly more intricate, giving rise to striking effects such as self‐focusing [1], soliton propagation [2], and high‐order harmonic generation [3, 4]. In third‐order NLO processes, two‐photon absorption (TPA) is especially noteworthy owing to its intrinsically low fluorescence background [5], excellent spatial resolution [6], and high energy density [7, 8]. Because a TPA chromophore simultaneously absorbs two photons, its excitation wavelength is effectively doubled relative to one‐photon excitation, thus easily enabling a shift from visible to near‐infrared (NIR) optical excitation. Compared with visible photons, NIR photons experience markedly reduced tissue scattering and autofluorescence, affording superior clarity and deeper penetration depths [9, 10, 11, 12]. Thus, TPA chromophores have been widely used in biological and molecular imaging [13, 14], photodynamic therapy (PDT) [15, 16], and other fields. Advanced fluorescent bioimaging technologies provide powerful tools for in‐depth analysis of biological processes and disease mechanisms [17, 18, 19, 20]. The Perturb‐Multimodal method developed by Zhuang's group combines imaging and sequencing techniques, utilizing multiplex fluorescence in situ hybridization or in situ sequencing to identify perturbations while integrating population genetic screening with single‐cell RNA sequencing data. This approach not only reveals the genetic basis of complex physiological phenomena but also significantly advances the construction of predictive models for cells and tissues. To date, various active materials, particularly inorganic nanostructures, have been applied in fluorescence imaging [21], including carbon nanotubes [22, 23], quantum dots (QDs) [24, 25], and others. However, these materials may cause bioaccumulation of toxicity, thus raising concerns about safety [26]. Recently, organic fluorescent probes, owing to their tunable electronic structure, ease of fabrication, and low biological toxicity, are becoming a more favorable alternative [27, 28, 29]. However, the synthesis of traditional organic monomeric fluorescent probes mainly relies on cumbersome covalent modification strategies [30, 31], and suffers no clear structural design principle and low yield, while improving the resolution of molecular imaging using these organic fluorescent probes currently still remains a significant challenge.

Cocrystal engineering, as a key paradigm for optoelectronic materials design, relies on non‐covalent interactions between donor (D) and acceptor (A) molecules to achieve D–A supramolecular ordering through cost‐effective solution processing [32, 33, 34, 35]. Such multicomponent systems combine the intrinsic properties of individual molecules with novel characteristics derived from molecular synergy [36]. Electronic orbital coupling induces hybridization between the donor HOMO and acceptor LUMO levels, leading to a narrowing bandgap and triggering a redshift in charge transfer (CT) absorption within the NIR region [37]. The precise control of cocrystal structures enables targeted optimization of photophysical properties, and their tunable size facilitates multifunctional applications and investigation of structure‐property relationships [38, 39, 40, 41, 42]: micron and nanoscale films are suitable for optoelectronic device integration, while nanoparticle configurations offer new avenues for the development of molecular fluorescence probes. More importantly, by adjusting the cocrystal structure, the strength of intermolecular CT can be tuned, significantly boosting the TPA performance [43]. With its tunable electronic structure and synergistic effects, cocrystal engineering provides an innovative strategy for the development of high‐performance TPA molecular fluorescence probes. However, traditional measurements of TPA cross‐sections are typically based on solution systems [44, 45, 46]. For solid‐state cocrystals, experimental characterization remains challenging, and current data are largely derived from theoretical calculations [27].

Herein, we choose 1,2,4,5‐Tetracyanobenzene (TCNB) as the electron acceptor and 9,10‐Bis[(E)‐2‐(pyridin‐4‐yl)vinyl]anthracene (BP4VA) as the electron donor, and construct a CT cocrystal BP4TC via the vapor diffusion method. Compared to the previously reported Bpe‐TCNB cocrystal system [43], the extended π‐conjugation of BP4VA shifts the maximum two‐photon excitation wavelength into the NIR tissue‐transparent window (700–1000 nm). To impart aqueous dispersibility, uniform organic nanoparticles (BP4TC‐NPs) are prepared via nanoprecipitation using the amphiphilic surfactant DSPE‐PEG 2000. Open‐aperture Z‐scan measurements provide the experimental determination of the TPA cross‐section for the cocrystal, with prominent absorption signals spanning the NIR‐I/II regions (700–1000 nm). The cross‐section values decrease monotonically with increasing excitation wavelength. First‐principles calculations demonstrate that BP4TC exhibits a stronger intermolecular CT capacity, thereby verifying its enhanced TPA ability. Furthermore, the TPA cross‐section of BP4TC was computed using the TDDFT (Time‐Dependent Density Functional Theory) approach. This further indicates that BP4TC is a potent two‐photon absorber. The optimal two‐photon excitation wavelength was identified at approximately 900 nm, and its robust two‐photon‐excited fluorescence imaging performance was experimentally validated in an in vitro cell model. The probe exhibits bright, high‐contrast intracellular emission and negligible cytotoxicity. The pronounced third‐order NLO response and superior imaging capability of these cocrystal nanoparticles enable them to be strong candidates for deep‑tissue imaging.

## 2 Results and Discussion

We initially design and synthesize a novel cocrystal through the vapor diffusion method, followed by crystal structure characterization via single‐crystal X‐ray diffraction and powder X‐ray diffraction. Subsequently, the fundamental optical properties of the cocrystal are investigated using UV–vis absorption spectroscopy and photoluminescence (PL) spectroscopy. The presence of intermolecular CT in the ground state of the cocrystal is corroborated through advanced techniques such as X‐ray photoelectron spectroscopy (XPS), solid‐state ^13^C nuclear magnetic resonance (^13^C‐NMR), and electron paramagnetic resonance (EPR) spectroscopy. To facilitate its application in biological imaging, we fabricate nanoscale cocrystal particles, and their excited‐state intermolecular CT behavior is analyzed using time‐resolved absorption spectroscopy. The TPA characteristics of the cocrystals, encapsulated within a surfactant, are validated through two‐photon confocal microscopy and Z‐scan measurements. Finally, we successfully captured two‐photon excited fluorescence cellular imaging of the nanoscale cocrystals using two‐photon confocal microscopy. All the experimental methodologies and theoretical insights are comprehensively reported in the Supporting Information.

### 2.1 Structure, Optical Properties, and Ground‐state Charge Transfer

**FIGURE 1:**
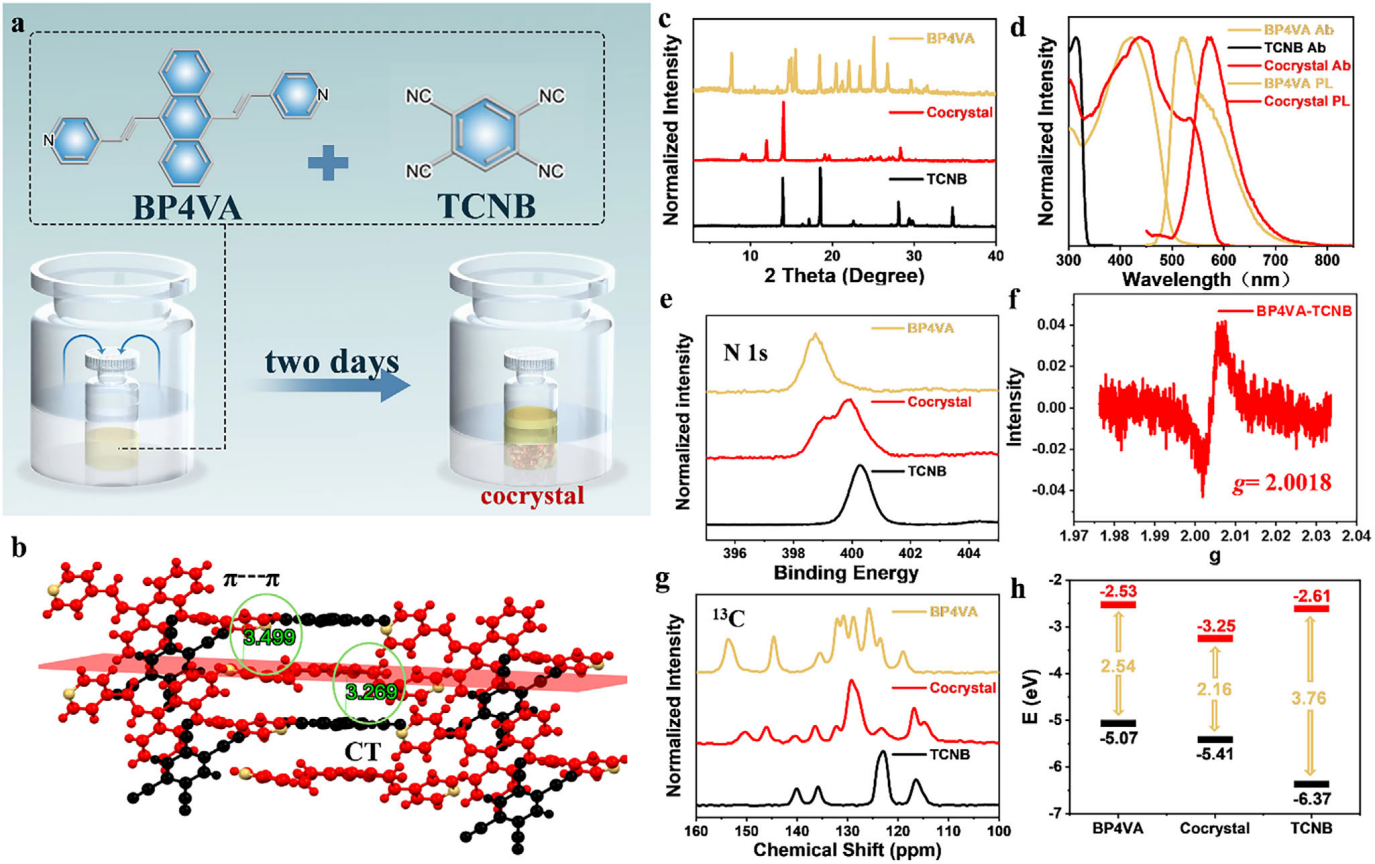
Cocrystal growth, morphology, and photophysical properties. (a) Single crystals of BP4TC are grown using the vapor diffusion method. (b) Intermolecular interactions in BP4TC. (c) PXRD patterns of BP4TC and the monomer. (d) Absorption and photoluminescence spectra of BP4TC and the monomer. (e) XPS spectrum of BP4TC. (f) Quantitative N‐vacancy spectra of BP4TC (g) solid‐state ^13^C‐NMR spectra; (h) Energy band structures of the monomer and the cocrystal.

We obtain a novel crystal with an extended conjugated system by means of vapor diffusion crystallization at the same donor‐acceptor (D‐A) concentration (Figure 1a), which we designate as BP4TC (CCDC No. 2356068). Detailed experimental procedures are outlined in Supporting Information. This new crystal exhibits a more robust conjugated system, compared to the Bpe‐TCNB system previously reported by our group [43], which exhibits further red‐shifted excitation and emission wavelengths. BP4TC crystallizes in the monoclinic P21/n space group with unit cell parameters of *a* = 18.5503 Å, *b* = 7.5700 Å, *c* = 19.8056 Å, *α* = 90°, *β* = 98.171°, *γ* = 90° (Table S1). This crystal belongs to a centrosymmetric space group, which precludes the occurrence of second‐harmonic generation (SHG), a competitive process that only arises in non‐centrosymmetric structures [47, 48]. Figure S2b illustrates that BP4TC adopts a mixed packing arrangement along the direction of intermolecular CT interactions, where the BP4VA and TCNB monomers form a two‐dimensional molecular plane through ─CH···N─ (2.239 Å) interactions. These planes then stack layer by layer through D‐A (3.269 Å), π–π (3.499 Å), and ─CH···N─ interactions between the donors (Figure S2b), resulting in a unique three‐dimensional molecular arrangement (Figure 1b). Figure 1c displays the powder X‐ray diffraction patterns for both the monomer and the cocrystal, clearly showing new diffraction peaks after co‐crystallization, indicative of the formation of a new substance.

We then performed a series of spectroscopic studies to determine the physicochemical properties of BP4TC. First, the absorption peak of BP4TC (Figure 1d) exhibits a significant redshift compared to its individual components, which is consistent with CT absorption characteristics. Next, steady‐state fluorescence spectroscopy reveals that, BP4VA shows a broad, structure‐less peak (FWHM: 45 nm) and a redshifted emission peak at 568 nm compared to the individual components (Figure 1d). BP4TC exhibits a large Stokes shift of 7519 cm^−^^1^, indicating that the fluorescence emission originates from a bimolecular excited‐state species rather than a single component, and significant recombination occurs in the excited state. The pronounced Stokes shift is advantageous for biological imaging, as it prevents self‐quenching and reduces background interference [49].

Figure 1e presents the XPS spectrum of the N1s region, where the binding energy of the nitrogen atom in the donor increases significantly, while the binding energy of the nitrogen in the acceptor decreases after self‐assembly. This observation is consistent with previously reported organic CT cocrystals [43]. Additionally, through quantitative EPR N‐vacancy testing (Figure 1f), the *g*‐value is determined to be very close to the *g*‐value of a free electron (2.0023), indicating the presence of unpaired electrons in the cocrystal, which further confirms the existence of CT in the ground state. Furthermore, we study the changes in the chemical environment of carbon atoms after co‐crystallization using solid‐state ^13^C‐NMR (Figure 1g). Notably, the peak at 123.0 ppm, representing the four carbon atoms in the phenyl ring of TCNB that are connected to the cyano group, shifts to a lower chemical shift value of 116.9 ppm after co‐crystallization, indicating an increase in the electron cloud density of the acceptor molecule. For BP4VA, the four carbon atoms in the pyridine ring adjacent to the nitrogen atom shift from 119.1 ppm to a higher chemical shift value of 123.4 ppm after co‐crystallization, while the eight carbon atoms on the wings of the anthracene ring, originally at 126.0 ppm, also shift to a higher chemical shift value of 129.2 ppm, indicating a decrease in electron cloud density for the donor molecule after co‐crystallization, which spreads to the anthracene ring and both pyridine rings. Additionally, the Raman spectra (Figure S5) shows that the BP4TC cocrystal exhibits a superposition of the two monomer peaks, such phenomenon is also observed in other CT cocrystals [50].

Next, we utilize UPS and UV–vis spectroscopy to obtain the highest occupied molecular orbital (HOMO) and lowest unoccupied molecular orbital (LUMO) energy levels for both the cocrystal and the monomer. First, based on the Tauc plot (Figure S6), the cocrystal exhibits a significantly smaller bandgap compared to the individual monomer, which results from electronic cloud rearrangement induced by intermolecular CT, leading to the formation of new molecular orbital energy levels. Subsequently, we determine the HOMO value from the secondary electron cutoff (*E*_cutoff_) and the Fermi edge (*E*_onset_) in the UPS spectrum. As shown in Figure 1h, the donor BP4VA molecule, with a large conjugation degree, exhibits a lower HOMO energy level. The HOMO energy level of the cocrystal is closer to that of the donor BP4VA, resulting in a narrower bandgap after co‐crystallization. Moreover, the LUMO energy levels of the donor and acceptor molecules are relatively close, satisfying the established energy level matching rule.

### 2.2 Cocrystal Nanoparticles and Excited‐State Charge Transfer

**FIGURE 2:**
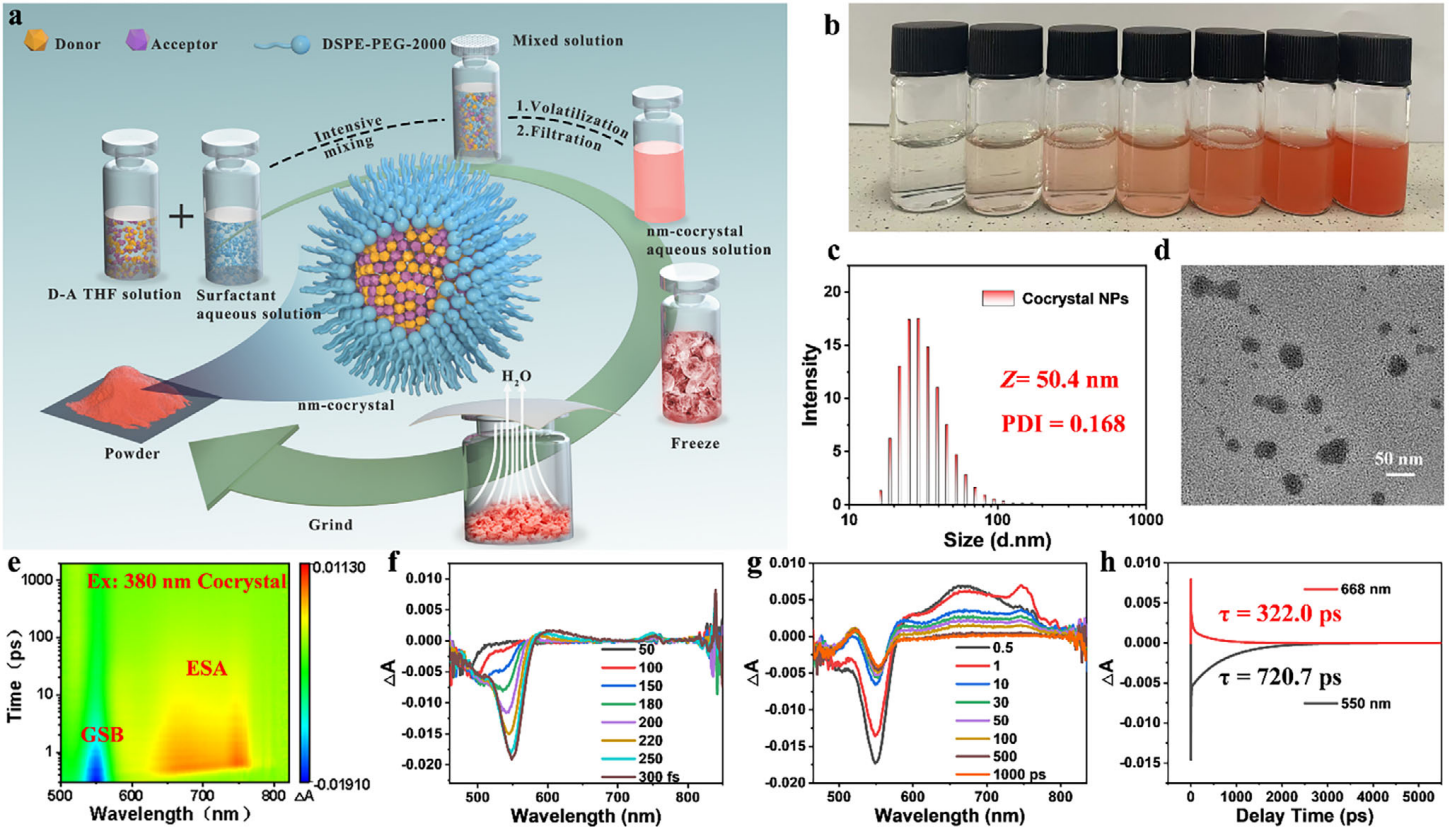
Cocrystal nanoparticle preparation and excited‐state charge transfer. (a) Schematic diagram of the preparation process of nanoscale organic charge transfer cocrystals. First, the tetrahydrofuran solvent is removed via nanoprecipitation, followed by freeze‐drying to remove water, resulting in the final nanoscale cocrystal powder that is ready for immediate use. (b) The figure shows, from left to right, pure deionized water, and BP4TC nanoparticle aqueous solutions at concentrations of 0.002, 0.006, 0.014, 0.020, 0.030, and 0.040 mg/mL. c) Particle size distribution of BP4TC nanoparticles. d) High‐resolution TEM image of the BP4TC nanoparticles. (e) Three‐dimensional transient absorption (TA) spectra of BP4TC nanoparticles. (f) & (g) fsTA spectra and their relationship with pump‐probe delay time. (h) Decay curves at 668 and 550 nm.

To apply this material in biological imaging, we fabricated nanoscale particles of the cocrystal, with the preparation process illustrated in Figure 2a. The detailed synthesis method is outlined in the Supporting Information. Importantly, we are able to prepare aqueous solutions of the nanoscale cocrystal at various concentrations, as shown in Figure 2b. Initially, Figure 2c presents the size of cocrystals (50.4 nm), exhibiting excellent dispersibility, and the corresponding Transmission Electron Microscope (TEM) image is shown in Figure 2d. Notably, the nanoscale cocrystal demonstrates exceptional TPA properties under 780 nm laser irradiation (Figure S8). This undoubtedly lays a solid foundation for its subsequent application in two‐photon‐excited fluorescence imaging. To investigate the properties of the nanoscale cocrystal aqueous solution in the excited state, we also performed femtosecond transient absorption (fsTA) spectroscopy measurements. As shown in Figure 2e, under 380 nm laser excitation, the cocrystal solution exhibits a distinct ground‐state bleaching (GSB) peak at 550 nm, while notable excited‐state absorption (ESA) peaks appear in the 620–760 nm wavelength range. The delayed appearance of these peaks relative to the GSB peak confirms our analysis. However, we did not detect the excited‐state absorption peak associated with TCNB•^−^ [43], which indirectly supports the notion that our material is a novel substance, not merely reflecting the characteristics of the D‐A components.

For comparison, we also measured fsTA spectra of the donor molecule BP4VA under the same excitation (Figure S9). BP4VA shows a broad GSB peak in the 470–545 nm range and a distinct ESA peak around 600 nm. Additionally, the molecule displays a prominent stimulated emission peak at 750 nm. In contrast to BP4VA, the cocrystal exhibits more complex behavior, likely due to electron transfer and recombination processes between the donor (BP4VA) and acceptor (TCNB). Notably, Figure 2h shows that the decay curve corresponding to the GSB peak exhibits a longer decay time, indicating a slower recovery of the ground‐state bleaching signal. This suggests that the electron remains in the excited state for a longer period, and the recombination process is relatively slow.

### 2.3 Z‐scan and Two‐photon Absorption Cross Section

**FIGURE 3:**
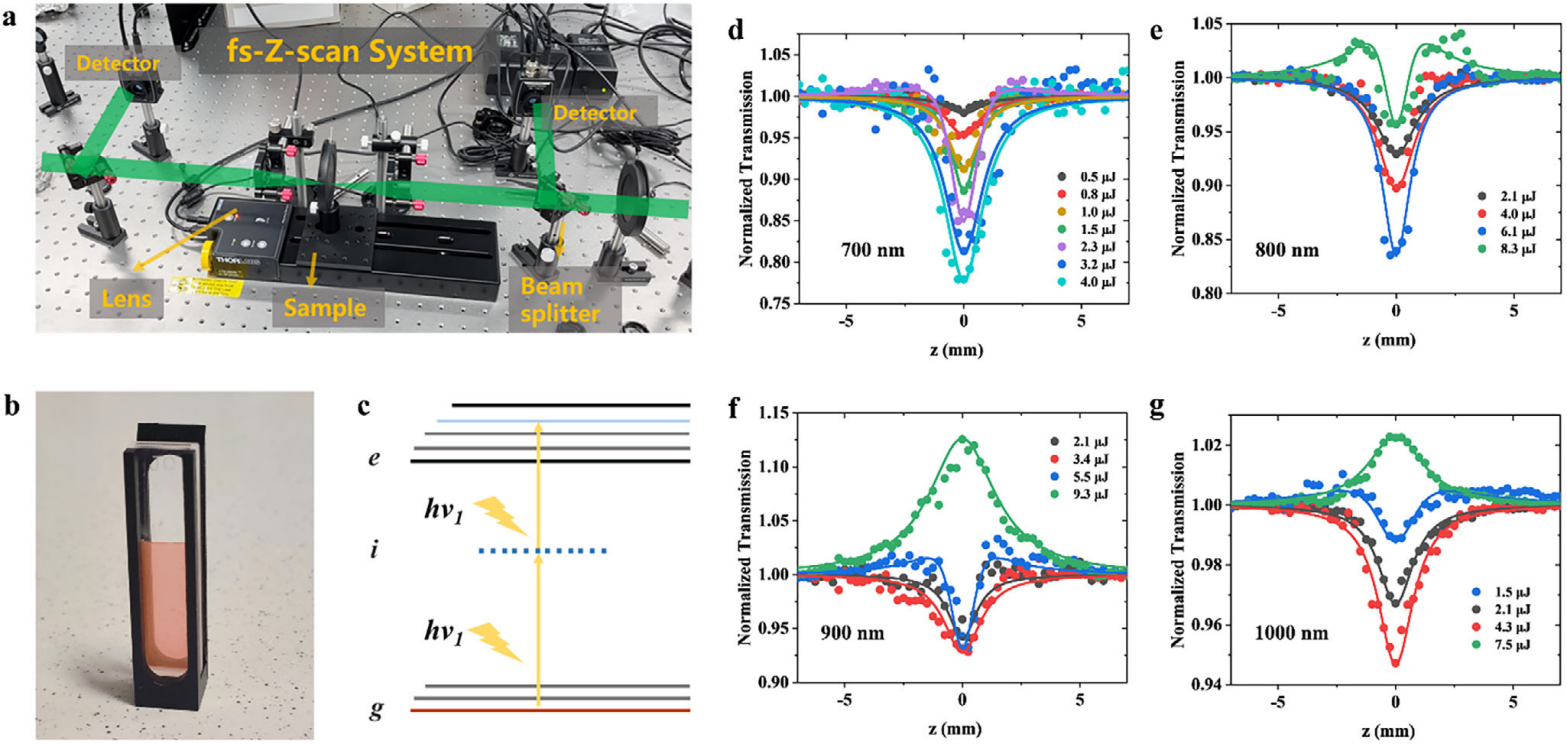
Two‐photon absorption characterized by open‐aperture Z‐scan. (a) Actual diagram of our home‐built open‐aperture Z‐scan setup. (b) Aqueous dispersion of BP4TC‐NPs contained in a 1‐mm quartz cuvette for OA Z‐scan measurements. (c) Diagram of TPA mechanism. Under the irradiation of a certain wavelength, electrons are excited to transition from the ground state to the excited state via an intermediate virtual state (g represents the initial ground state, i represents the intermediate state, and e represents the excited state). OA Z‐scan Data of BP4TC Nanoparticles (4.983 × 10 ^−4^ mol/L) under 200 fs laser pulses: (d) 700 nm with different energies of 0.5, 0.8, 1.0, 1.5, 2.3, 3.2, and 4.0 µJ. (e) 800 nm with different energies of 2.1, 4.0, 6.1, and 8.3 µJ; (f) 900 nm with different energies of 2.1, 3.4, 5.5, and 9.3 µJ. (g) 1000 nm with different energies of 1.5, 2.1, 4.3, and 7.5 µJ.

The cocrystal material is fabricated into nanoparticles and dispersed in water, enabling the use of Z‐scan technology to evaluate the material's NLO properties. We employed open‐aperture (OA) Z‐scan measurements with a pulse width of 200 fs at various wavelengths. Specific experimental conditions and setup details are outlined in the SI. As shown in Figure 3d, when the sample is moved toward the focus to increase the incident irradiance, the normalized transmittance (*T*_norm_) of the cocrystal nanoparticles at 700 nm decreases significantly at input intensities (*I*_0_) of 0.5, 0.8, 1.0, 1.5, 2.3, 3.2, and 4.0 µJ, indicating an NLO response generated by reverse saturation absorption (RSA). A similar trend is observed at 800, 900, and 1000 nm; however, at 900 nm with *I*_0_ = 9.3 µJ and at 1000 nm with *I*_0_ = 7.5 µJ, an opposite trend (peak‐valley) is observed, indicating an NLO response due to saturation absorption (SA). Notably, at all wavelengths, an increase in *I*_0_ leads to an enhanced NLO response generated by RSA, with the maximum response observed at *I*_0_ = 4 µJ at 700 nm. Considering the π‐conjugated D−A structure of the cocrystal, we infer that intermolecular CT plays a significant role in the NLO response in the NIR region [51]. This mechanism has been reported in literature for some organic‐inorganic hybrid materials. Then, we extracted the TPA coefficient (β) by performing numerical fitting of the propagation process, and the TPA cross‐section (δ_2PA_) was derived from β using the corresponding formula. As shown in Table 1, the δ_2PA_ values of the cocrystal at excitation wavelengths of 700, 800, 900, and 1000 nm are 272, 85, 56, and 35 GM, respectively. As the excitation wavelength increases, the TPA cross‐section value decreases, and the difference in δ_2PA_ between 800 nm and 700 nm is significant, leading us to infer that the peak of TPA occurs around 700 nm.

**TABLE 1:** Fitting results from the Z‐scan measurements and the corresponding calculated TPA cross‐sections (The cocrystal concentration is 4.983 × 10^−^^4^ mol L^−^^1^).

Excitation wavelength (nm)	Nonlinear absorption coefficient β_avg_ (m/W)	Experimental TPA cross‐section δ_2PA_ (GM)	Theoretical TPA cross‐section δ_2PA_ (GM)
700	2.872×10^−14^	272	4.2
800	1.024×10^−14^	85	3.1
900	7.610×10^−15^	56	3.4
1000	5.303×10^−15^	35	13.9

### 2.4 First‐principles Calculations

**FIGURE 4:**
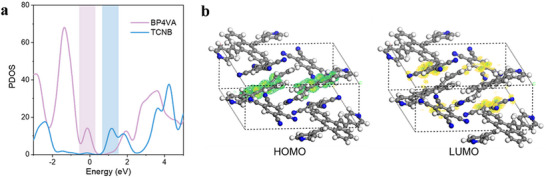
First‐principles calculations of BP4TC. (a) Partial density of states (PDOS) of cocrystal BP4TC. (b) Highest occupied molecular orbital (HOMO) and lowest unoccupied molecular orbital (LUMO).

The first‐principles calculations were performed on partial density of states (PDOS) as well as frontier orbitals using CASTEP package based on the density‐functional theory (DFT) [52, 53, 54]. The PDOS and frontier orbital projections (Figure 4) demonstrate that the top of the valence band of cocrystal originates from BP4VA, while the bottom of the conduction band is mainly contributed by TCNB. The calculated bandgap of cocrystal is decreased compared to both monomers (Table S5). The results are basically consistent with the UPS and UV–vis data disscussed above. In addition, the dipole moments of the ground (*µ_g_*) and the first excited states (*µ*_e_) for the cocrystal are calculated using the Gaussian 09 package [55]. The B3LYP functional, along with the 6–31G(d) basis set was chosen [53, 56, 57, 58, 59]. For BP4TC, the difference in dipole moments between the ground and excited states (*Δµ = µ_e_ – µ_g_*) is 47.06, which is much higher than that of other TCNB‐based cocrystals (Table S3). This discrepancy may be attributed to the extended conjugation in the donor molecule BP4VA, which enhances intermolecular CT interactions. Since *Δµ* is proportional to the TPA capacity [60], it can be concluded that BP4TC exhibits strong TPA performance [43].

Additionally, to more accurately describe the TPA property of BP4TC [61], the TDDFT (Time‐Dependent Density Functional Theory) method was employed to calculate the TPA cross‐section, utilizing the CAM‐B3LYP functional and the def2‐TZVP basis set. As presented in Table 1, the theoretically computed TPA cross‐sections were determined to be 4.2, 3.1, 3.4, and 13.9 GM at excitation wavelengths of 700, 800, 900, and 1000 nm, respectively. Notably, the calculated results not in the Table show that the cross section rises to 272 GM at 580 nm, so there is an overall blue shift in the calculated spectra compared to the measurements. The exact origin of this discrepancy remains unclear; nevertheless, potential contributing factors may include size effects of the investigated samples and the influence of solvent effects. Based on the integrated experimental and theoretical computational results, we can arrive at the conclusion that BP4TC is a strong two‐photon absorber at wavelengths in the 700 nm range.

### 2.5 Two‐photon Absorption Characteristics and Fluorescence Imaging of human Lung Cancer Cells

**FIGURE 5:**
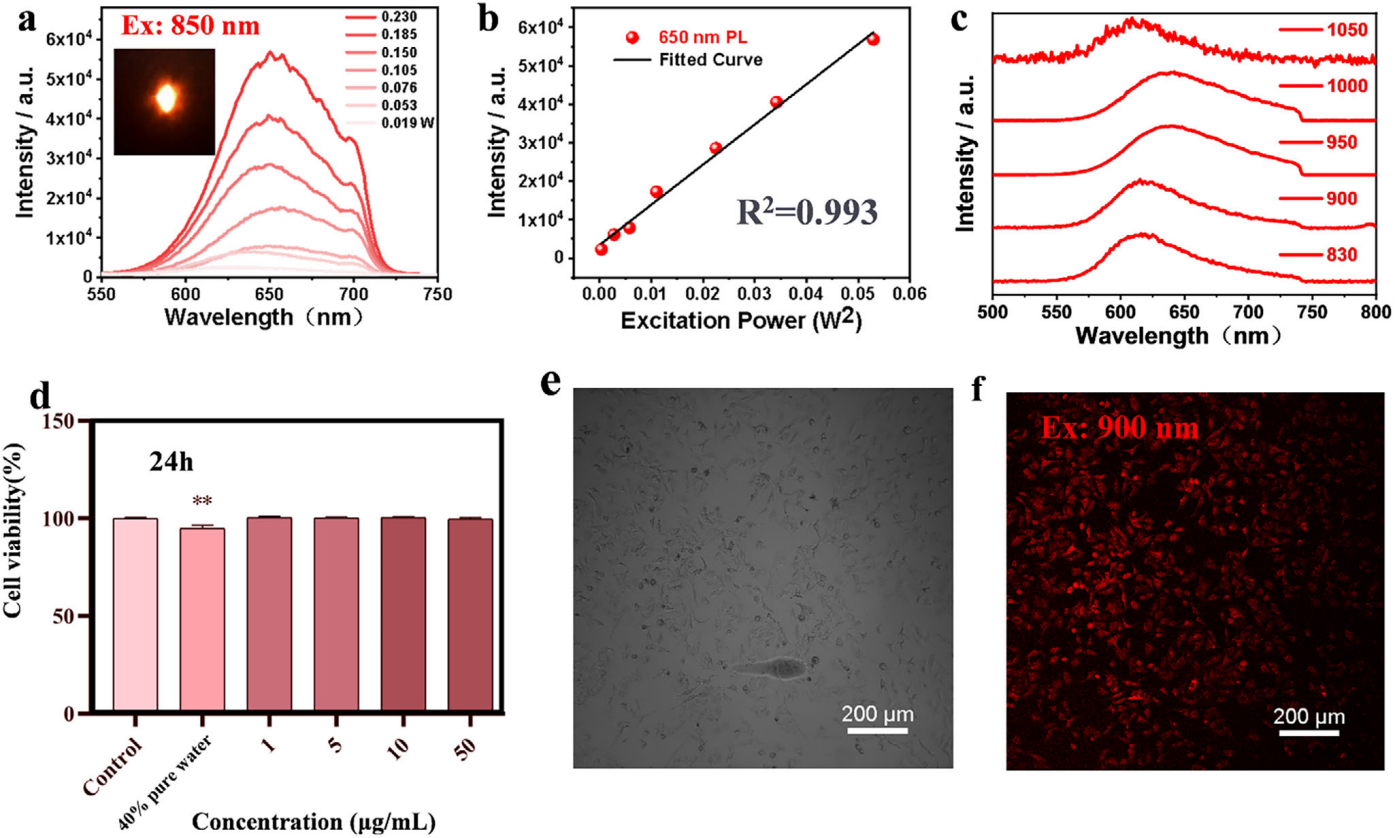
Two‐photon absorption of BP4TC and fluorescence imaging of BP4TC‐NPs in human cells. (a) Two‐photon absorption spectra of BP4TC cocrystal under 850 nm excitation, with an inset image showing the laser focused on the powder sample at the focal point. (b) The relationship between the emitted light intensity and the square of the excitation light power (using 650 nm as the optimal emission wavelength). (c) Two‐photon excited fluorescence spectra of BP4TC in the 830–1050 nm wavelength range. (d) Cytotoxicity tests on A549 cells at different mass concentrations for BP4TC‐NPs aqueous solution. (e) Bright‐field image of human lung cancer A549 cells. (f) Two‐photon excited fluorescence (TPEF) image of A549 cells incubated with BP4TC cocrystal nanoparticles under 900 nm excitation, with an image signal‑to‑noise ratio(SNR) of 33.84:1.

As shown in Figure 5a, under 850 nm excitation, we successfully detect the TPA signal of the cocrystal, which intensifies with increasing excitation power. Notably, the fluorescence spectra under two‐photon excitation exhibit a peak emission at 650 nm, which shows a significant redshift compared to the one‐photon optimal emission peak at 620 nm, making it more suitable for applications in molecular imaging. By fitting the relationship between the emitted light intensity and excitation power, we find that the emitted light intensity exhibits a linear dependence on the square of the excitation power (Figure 5b). To investigate the maximum excitation wavelength, we use lasers of different wavelengths as excitation sources to detect the two‐photon excited fluorescence signal (Figure 5c). Due to the instrumental limitations, we have only experimentally confirmed the TPA response at the 1000 nm wavelength range (Figure S7). Given that the UV‐vis absorption cutoff of the BP4TC is around 600 nm (Figure 1d), we believe that this material can extend its excitation range to approximately 1200 nm, thereby covering the entire NIR‐I region and part of shortwave infrared (SWIR) region. Before performing two‐photon confocal imaging of A549 cells (human lung cancer cells), the cytotoxicity of the cocrystal nanoparticles was evaluated. As shown in Figure 5d, the cocrystal nanoparticle aqueous solution exhibits low cytotoxicity across a wide range of concentrations, from 1 to 50 µg/mL. To confirm that the fluorescence signal originates from the cocrystal rather than the monomer, we prepared BP4VA nanoparticles and performed fluorescence spectroscopy (Figure S4). The emission peak appears in the yellow‑‐green region, while the characteristic red emission from the cocrystal phase is absent in the spectrum. The surfactant DSPE‐PEG‐2000, employed for cocrystal encapsulation, exhibits no intrinsic fluorescence and therefore does not contribute to or interfere with the fluorescence signal from the cocrystal [43]. Furthermore, the stability of BP4TC nanoparticles was verified under physiological conditions (Table S4). The findings confirm that the nanoparticles exhibit favorable stability. Subsequently, we incubate the nanoscale cocrystal aqueous solution with human lung cancer cells for 15 min, and observe live cells using two‐photon confocal microscopy at the optimal excitation wavelength of 900 nm, as shown in Figure 5f. In comparison with the bright‐field image of the human lung cancer cells (Figure 5e), we can clearly observe that the cocrystal nanoparticles successfully penetrate the cell membrane and also enter the cells, thereby demonstrating the material's excellent two‐photon excited fluorescence imaging capability.

## 3 Conclusions

In conclusion, we have employed a vapor diffusion strategy to construct charge transfer cocrystal BP4TC from BP4VA (donor) and TCNB (acceptor). Single‐ and powder‐X‐ray diffraction, together with UV–vis absorption, steady‐state photoluminescence, X‐ray photoelectron spectroscopy, electron‐paramagnetic resonance, and solid‐state ^1^ ^3^C‐NMR reveal a D–A π‐stack and pronounced ground‐state intermolecular charge transfer, suggesting strong third‐order nonlinear optical behaviour. Nanoprecipitation with DSPE‐PEG 2000 yields stable BP4TC nanoparticles (≈50 nm). Femtosecond transient‐absorption spectroscopy shows an elongated charge transfer lifetime in the excited state, providing a favourable kinetic window for two‐photon absorption (TPA) processes. Open‐aperture Z‐scan measurements afford, for the first time, experimentally determined TPA cross‐sections for an organic cocrystal: prominent reverse‐saturable and saturable absorption signals span 700–1000 nm, with δ values of 35–272 GM that diminish monotonically with increasing wavelength. First‐principles calculations demonstrate that BP4TC exhibits a stronger intermolecular charge transfer capacity, thereby verifying its enhanced TPA ability. Furthermore, the TPA cross‐section of BP4TC was computed using the TDDFT approach. This further indicates that BP4TC is a potent two‐photon absorber. Under 850 nm excitation the nanoparticles emit intense two‐photon‐excited fluorescence, and 900 nm is identified as the optimal excitation wavelength for bio‐applications. In vitro studies with A549 cells demonstrate bright, high‐contrast intracellular fluorescence and negligible cytotoxicity, confirming BP4TC‐NPs as a viable biological multiphoton imaging probe. By integrating broadband NIR TPA response with benign biological profiles, BP4TC establishes a new material archetype for organic cocrystals in multiphoton bio‐imaging and NIR photonics, while providing an experimental blueprint for overcoming the long‐standing challenge of quantifying cocrystal TPA performance.

## 4 Experimental Section

### 4.1 Reagents and Materials

All starting materials were purchased from commercial sources and used without further purification, unless mentioned otherwise. Three distinct methods were employed to prepare BP4TC cocrystals. Gram‐scale powders were synthesized by solvent‐assisted mechanical grinding of BP4VA and TCNB precursors followed by solvent evaporation. Single crystals were grown via vapor diffusion using dichloromethane/n‐hexane systems. Nanoparticles were produced through nanoprecipitation with DSPE‐PEG 2000 surfactant, with subsequent freeze‐drying to ensure colloidal stability.

### 4.2 Z‐Scan Measurements

The nonlinear optical properties of BP4TC‐NPs were systematically investigated using a home‐built open‐aperture Z‐scan system. The setup employed a tunable fs‐laser (200 fs, 750 kHz) with automated sample translation (<5 µm precision). Samples satisfied the thin‐sample approximation (L << z_0_) in 1‐mm quartz cuvettes at 25 ± 0.5 °C. Nonlinear absorption coefficients were extracted by fitting data to the propagation equation,

$$\frac{dI\left(z\right)}{dz}=-\alpha \left(I\right)I$$

where *I* denotes the incident intensity, *z* represents the propagation distance within the sample. The absorption coefficient α (*I*) can be described as:

$$\alpha \;\left(I\right)=\alpha_{0}+\beta I$$

where α_0_ and *β* are the linear and non‐linear absorption coefficients, respectively. When two‐photon absorption (TPA) dominates, *β* stands for TPA coefficient that can be obtained through the OA Z‐scan data.

Formula for calculating the two‐photon absorption cross section, δ_2PA_ is derived from β using the following expression, in which *N*_A_ denotes Avogadro's number, c is the sample concentration, and hν corresponds to the photon energy of the excitation light.

$$\delta_{2PA}=\frac{h\nu \beta }{N_{A}c}\;$$

### 4.3 Theoretical Calculations

The CA‐PZ generalization under the local density approximation (LDA) was chosen for the exchange‐correlation functional. The dispersion correction for DFT (DFT‐D) is adopted to deal with the van der Waals interactions. The ultrasoft pseudopotentials are used for all elements, in which the H 1s, C 2s^2^2p^2^, and N 2s^2^2p^3^ electrons serve as valence electrons. The cutoff energy of the plane‐wave was set to 280 eV, and the k‐point of the Monkhorst‐Pack in the Brillouin zone was set to 1 × 3 × 1 [62]. The dipole moments of the ground state (*µ_g_*) and the first excited state (*µ_e_*) of the cocrystal were computed with the Gaussian 09 software package, employing the B3LYP functional and the 6–31G(d) basis set.

### 4.4 Statistical Analysis

For the size analysis of cocrystal nanoparticles, normalization was the sole preprocessing step applied. After normalization, the y‐axis in the representations was uniformly denoted as “Normalized Intensity.”

## Author Contributions

W. Zhu conceived the project and supervised the research work. L. Hao conceived the experimental plans, prepared the samples, and performed the spectroscopic experiments. Y. Ni performed experiments, prepared the draft, and revised the paper. J. Huang conducted Z‐scan measurements and fitted the data. Y. Wang and X. Wang performed cell fluorescence imaging experiments. F. Liu, L. Kang, and Z. Lin performed and analyzed the quantum chemical DFT calculations. All the authors discussed the results, wrote, and approved the final manuscript and SI.

## Conflicts of Interest

The authors declare no conflicts of interest.

## Supporting information




**Supporting File**: advs73848‐sup‐0001‐SuppMat.docx.

## Data Availability

The authors declare that all data supporting the findings of this study are available within the paper and Supplementary Information files. Source data are provided with this paper.
