# Supplementary material for: Near‐Infrared Cocrystal Nanofluorophore with Enhanced Two‐Photon Absorption Cross Sections
Source: Adv Sci (Weinh). 2026 Jan 15;13(17):e23319. doi: 10.1002/advs.202523319 (PMC13042663; doi:10.1002/advs.202523319)
Supplement: Supplementary file 1 — Supporting File: advs73848‐sup‐0001‐SuppMat.docx. [file ADVS-13-e23319-s001.docx]

**Supporting Information**

**Near-Infrared Cocrystal Nanofluorophore with Enhanced Two-Photon Absorption Cross Sections**

Liangmeng Hao^1,5^, Ying Ni^1,5^, Jiawei Huang^2^, Yucheng Wang^3^, Fan Liu^4^, Xu Wang^3^, Lei Kang^4^, Zheshuai Lin^4^, Weigang Zhu^1^*

^1^ State Key Laboratory of Advanced Materials for Intelligent Sensing, Key Laboratory of Organic Integrated Circuits, Ministry of Education, Tianjin Key Laboratory of Molecular Optoelectronic Sciences, Department of Chemistry, School of Science, Tianjin University, Tianjin 300072, China

^2^ Songshan Lake Materials Laboratory, Dongguan 523808, China

^3^ College of Chemistry, Chemical Engineering and Materials Science, Key Laboratory of Molecular and Nano Probes, Ministry of Education, Collaborative Innovation Center of Functionalized Probes for Chemical Imaging in Universities of Shandong, Shandong Normal University, Jinan 250014, China

^4^ Functional Crystal Laboratory, Technical Institute of Physics and Chemistry, Chinese Academy of Sciences, Beijing 100190, China

^5^ These authors contributed equally: Liangmeng Hao, Ying Ni

*E-mail: w_zhu10@tju.edu.cn

**Table of contents**

[**1. Reagents and materials S3**](#_Toc217757992)

[**2. Preparation of powder BP4TC charge transfer cocrystal powders S5**](#_Toc217757993)

[**3. Single crystal BP4TC growth via the vapor diffusion method S6**](#_Toc217757994)

[**4. Preparation of organic charge transfer cocrystal nanoparticles S7**](#_Toc217757995)

[**5. Single Crystal X-ray Diffraction (SXRD) S8**](#_Toc217757996)

[**6. Solid-state ^13^CNMR Spectroscopy S10**](#_Toc217757997)

[**7. UV-vis absorption, Photoluminescence spectroscopy S12**](#_Toc217757998)

[**8. Electron Paramagnetic Resonance (EPR) Spectroscopy S13**](#_Toc217757999)

[**9. Raman spectrum S14**](#_Toc217758000)

[**10. Ultraviolet Photoelectron Spectroscopy (UPS) S15**](#_Toc217758001)

[**11. X-ray photoelectron spectroscopy (XPS) S16**](#_Toc217758002)

[**12. Two photon absorption property S17**](#_Toc217758003)

[**13. Two-photon excited emission spectra S18**](#_Toc217758004)

[**14. Fs transient absorption (TA) spectroscopy S19**](#_Toc217758005)

[**15. Z-Scan Nonlinear Optical Absorption Measurements S20**](#_Toc217758006)

[**16. Two photon excited fluorescence imaging of A549 S22**](#_Toc217758007)

[**17. Stability assessment of BP4TC-NPs in physiological conditions S25**](#_Toc217758008)

[**18. First-principles calculations S26**](#_Toc217758009)

[**References S27**](#_Toc217758010)

# 1. Reagents and materials

Donor BP4VA 9,10-Bis[(E)-2-(pyridin-4-yl)vinyl]anthracene, CAS: 113308-38-6, 98%) was purchased from Bide Pharmatech Ltd.; Acceptor TCNB (1,2,4,5-Tetracyanobenzene, CAS: 712-74-3, 98%) and surfactant DSPE-PEG-2000 (CAS: 147867-65-0, 99%) were purchased from Tianjin Heowns OPDE Technologies, LLC; Tetrahydrofuran (HPLC) and dichloromethane (HPLC) were purchased from Meryer (Shanghai) Biochemical Technology Co. All of the chemicals were directly used without further purifications. Deionized water (18.2 MΩ·cm^-1^) was made by a Mill-Q water purification machine (ELGA, Purelab ultra, model: ULXXXGEM2, Tianjin Key Laboratory of Molecular Optoelectronic Sciences).


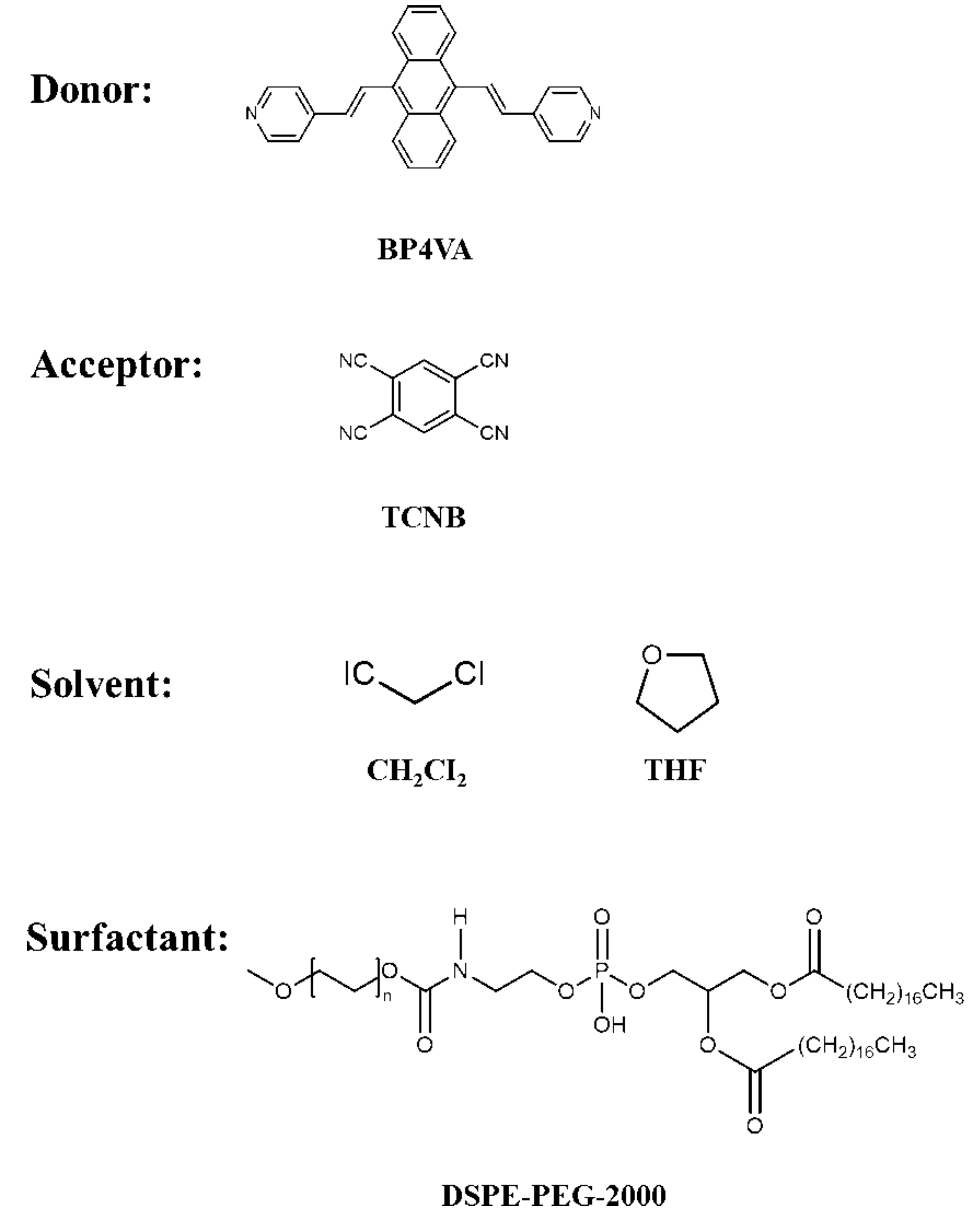


**Figure S1.** The chemical structures of donor, acceptor, solvent, and surfactant.

# 2. Preparation of powder BP4TC charge transfer cocrystal powders

Yellow BP4VA (1.78 mmol) and white TCNB (1.78 mmol), with a combined mass of 1 g, were combined in a mortar. Following the addition of dichloromethane solvent (10 mL), a red suspension formed rapidly. The mixture was then continuously triturated until the organic solvent had fully evaporated, yielding red BP4TC charge-transfer cocrystal powders as the final product.

# 3. Single crystal BP4TC growth via the vapor diffusion method

Since direct solvent evaporation failed to yield single crystals, we turned to the vapor diffusion method, following Garner et al^[1]^. Dichloromethane (good solvent) and n‑hexane (poor solvent/antisolvent) were used based on solubility differences. The controlled diffusion of n‑hexane into the solution lowered solubility gradually, enabling ordered nucleation and growth. This approach allowed us to acquire high‑quality crystals and resolve the cocrystal structure.

Specific operation: BP4VA (1 mg) and TCNB (0.5 mg) were entirely dissolved in 1.5 mL of dichloromethane using an open glass vial. This uncapped vial was then positioned inside a larger, sealed chamber holding 2 mL of n-hexane. The assembly remained undisturbed at ambient temperature for about two days, yielding red crystals deposited on the vial base.

# 4. Preparation of organic charge transfer cocrystal nanoparticles

For the preparation of nanoparticles, we selected DSPE-PEG-2000 (Distearoylphosphatidylethanolamine-Methoxy Polyethylene Glycol-2000) to encapsulate the organic cocrystal. As an amphiphilic molecule, DSPE-PEG-2000 has been widely used in nanoparticle formulation^[2–4]^. During nanoparticle formation, the hydrophobic DSPE segment tends to wrap around the hydrophobic core, while the hydrophilic PEG chains extend into the aqueous phase. This facilitates efficient encapsulation of our cocrystal material and enhances its solubility in aqueous solutions. Furthermore, we did not explore other surfactants such as P123 (poly (ethylene oxide)–poly (propylene oxide)–poly (ethylene oxide)) and Pluronic F-127 (poly (ethylene glycol)-block-poly (propylene glycol)-block-poly (ethylene glycol))^[5,6]^, as DSPE-PEG-2000 already met our encapsulation requirements.

Specific operation: A solution was prepared by dissolving BP4VA (0.0356 mmol) and TCNB (0.0356 mmol) in 5 mL of tetrahydrofuran. This solution was then rapidly injected into 10 mL of deionized water containing DSPE-PEG-2000 (20 mg). The resulting mixture was vigorously stirred at room temperature for 10 hours to ensure complete evaporation of the organic solvent, yielding an aqueous dispersion of organic charge-transfer cocrystal nanoparticles. Although the freshly formed nanoparticles displayed sizes under 100 nm, they underwent irreversible aggregation upon prolonged storage. To counteract this instability, the aqueous dispersion was immediately frozen in a standard refrigerator freezer overnight. The sample was subsequently freeze-dried, producing solid-state organic charge-transfer cocrystal nanoparticles. This method allowed for the on-demand preparation of non-aggregated nanoparticles with preserved colloidal stability.

# 5. Single Crystal X-ray Diffraction (SXRD)


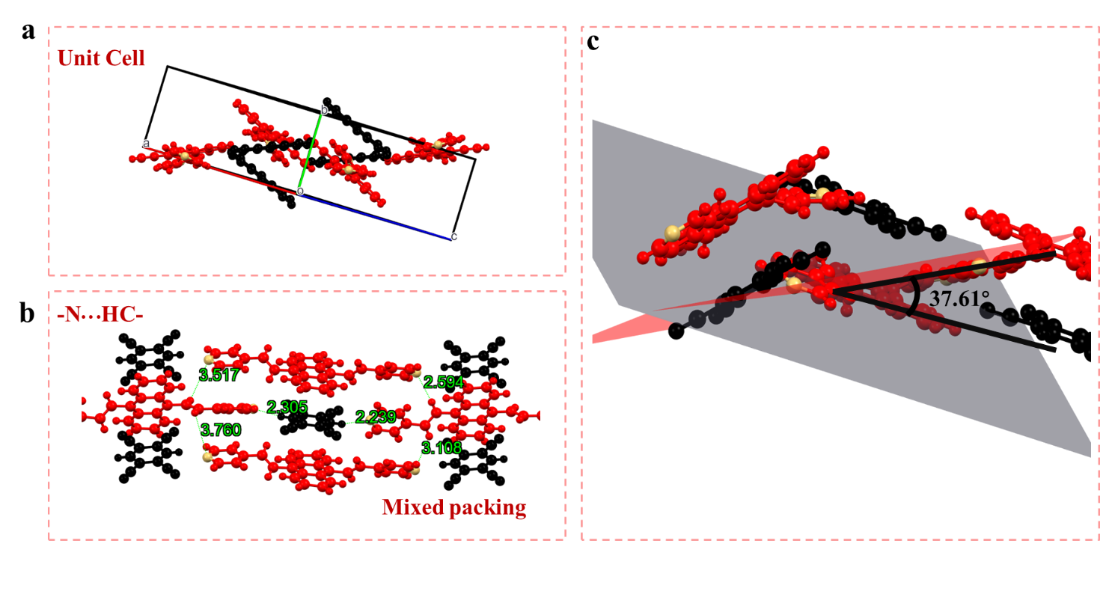


**Figure S2.** Single crystal structure of BP4TC.

|  | BP4TC |
| --- | --- |
| CCDC number | 2356068 |
| Empirical formula | C_38_H_22_N_6_ |
| Formula weight / g·mol^-1^ | 562.61 |
| Temperature / K | 130.00(10) |
| Crystal system | monoclinic |
| Space group (number) | P2_1_/n |
| a/ Å | 18.5505(3) |
| b/ Å | 7.5700(2) |
| c/ Å | 19.8056(3) |
| α/ ° | 90 |
| β/ ° | 98.171(2) |
| γ/ ° | 90 |
| Volume [Å^3^] | 2753.01(10) |
| Z | 4 |
| ρcalc /g·cm^−3^] | 1.357 |
| μ /mm^−1^ | 0.649 |
| F (000) | 1168 |
| Crystal size / mm^3^ | 0.03 × 0.03 × 0.1 |
| Crystal colour | red |
| Crystal shape | needle |
| Radiation | Cu Kα (λ=1.54184 Å) |
| 2θ range / ° | 6.11 to 151.81 (0.79 Å) |
|  | −18 ≤ h ≤ 22 |
| Index ranges | −9 ≤ k ≤ 9 |
|  | −24 ≤ l ≤ 24 |
| Reflections collected | 21489 |
|  | 5390 |
| Independent reflections | Rint = 0.0285 |
|  | Rsigma = 0.0289 |
| Data / Restraints / Parameters | 5390 / 0 / 397 |
| Absorption correction T_min_/ T_max_ (method) | 0.8650 / 1.0000 |
|  | (multi-scan) |
| Goodness-of-fit on F2 | 1.12 |
| Final R indexes [I≥2σ (I)] | R1 = 0.0448 |
|  | wR2 = 0.1227 |
| Final R indexes [all data] | R1 = 0.0520 |
|  | wR2 = 0.1265 |
| Largest peak/hole / e Å^−3^ | 0.33 /−0.20 |

**Table S1**. The crystal data and structure refinement for BP4TC.

# 6. Solid-state ^13^CNMR Spectroscopy

Solid-state nuclear magnetic resonance carbon (^13^CNMR) spectra were collected on a JEOL JNM ECZ600R spectrometer (600 MHz) at School of Science, Tianjin University. Measurements utilized a 15 kHz CPMG pulse sequence with parameter configurations of 80-second relaxation intervals and 0.1-microsecond pulse widths.

**Sample preparation:** Single-component powders were commercially sourced from chemical suppliers, whereas co-crystallized materials required pulverization of laboratory-grown single crystals prior to analysis.

**Data Processing:** Spectral datasets from solid-state NMR experiments underwent processing in Mestrelab's Mestrenova software (v14.0.0-23239). The free induction decay was initially cropped to suppress noise interference. Resolution enhancement was subsequently achieved through zero-filling operations before final manual optimization of spectral baselines.


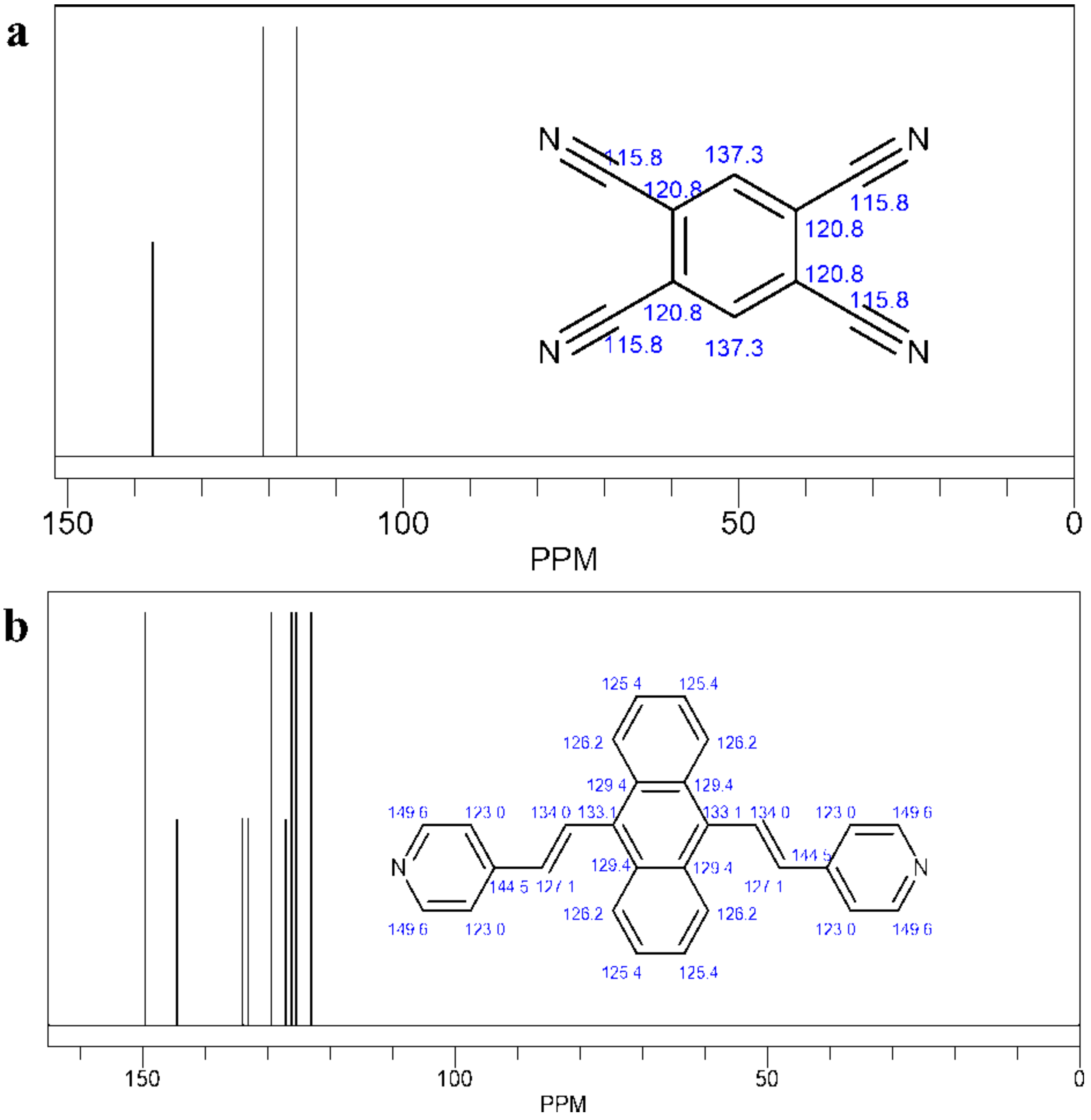


**Figure S3.** The predicted ^13^C-NMR spectra of (a) TCNB, (b) BP4VA using ChemDraw software (Level: Professional; Version: 19.0.0.22).

# 7. UV-vis absorption, Photoluminescence spectroscopy

**UV-vis absorption:** Absorption spectra in the ultraviolet-visible range were recorded on a Shimadzu UV 3600Plus spectrophotometer at the Tianjin Key Laboratory of Molecular Optoelectronic Sciences. Reflectance measurements were performed by depositing powdered samples onto BaSO₄ substrates. Following baseline correction, spectral data acquisition was initiated.

**Photoluminescence (PL) spectroscopy:** Photoluminescence spectral data were recorded on a Hitachi F-7000 FL spectrophotometer at the Tianjin Key Laboratory of Molecular Optoelectronic Sciences. The measurement protocol involved three sequential phases: initial fluorescence mode selection to identify optimal emission peaks using fixed excitation wavelengths, followed by excitation mode activation with locked emission maxima to determine optimal excitation wavelengths, culminating in final fluorescence spectrum acquisition at the established optimal excitation parameters. Single-component powders were procured commercially from chemical suppliers, whereas co-crystalline materials underwent mechanical processing of laboratory-grown crystals prior to characterizations.


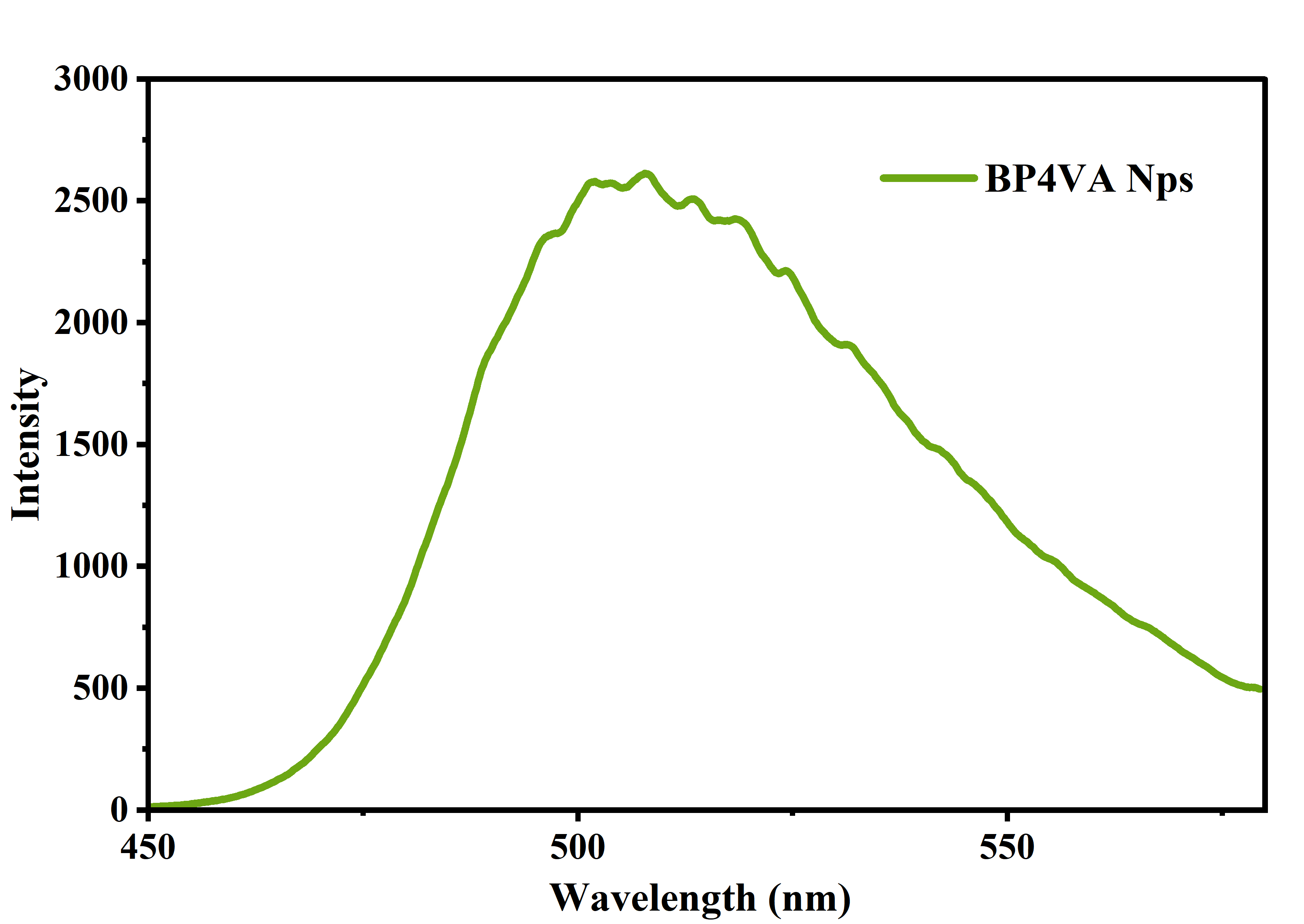


**Figure S4**. Fluorescence Spectrum of BP4VA Nanoparticles.

# 8. Electron Paramagnetic Resonance (EPR) Spectroscopy

Quantitative electron paramagnetic resonance analysis of nitrogen vacancies was performed at ambient temperature under dark-field conditions utilizing a Bruker EMXplus-6/1 spectrometer. Co-crystalline powders produced by mechanical pulverization were directly analyzed via EPR without further processing.

# 9. Raman spectrum


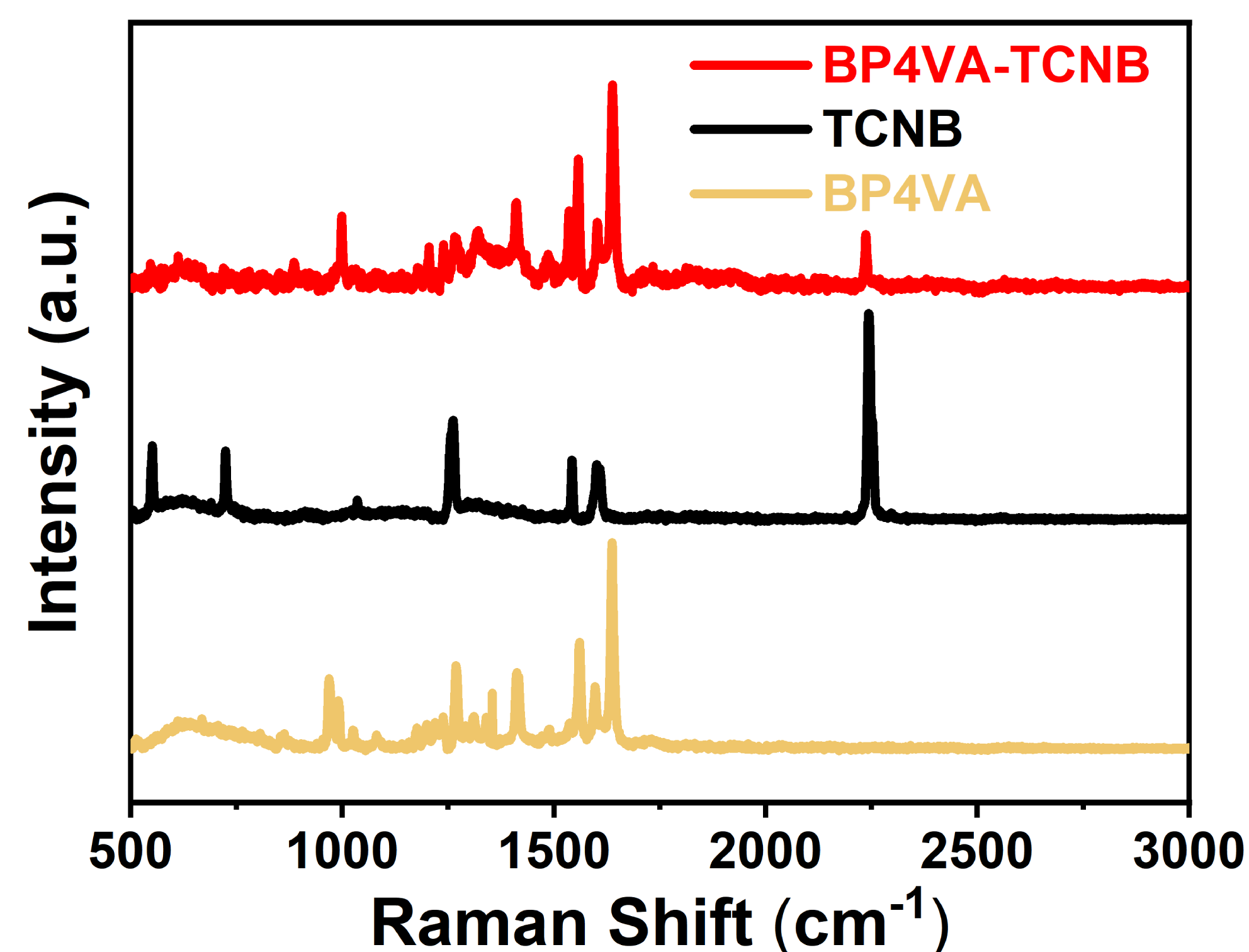


**Figure S5.** The Raman spectrum of TCNB, BP4VA, and BP4VA-TCNB.

# 10. Ultraviolet Photoelectron Spectroscopy (UPS)

All the experimental procedures and determinations of highest occupied molecular orbital (HOMO) energies were performed according to previously established methodologies.^[7]^ Single-component powders were commercially procured from chemical suppliers, whereas co-crystalline specimens underwent pulverization of laboratory-grown crystals before analysis. Cryogenic cooling with liquid nitrogen was implemented prior to measurements to suppress thermal noise. Samples were prepared by compression onto conductive-adhesive-coated aluminum substrates.


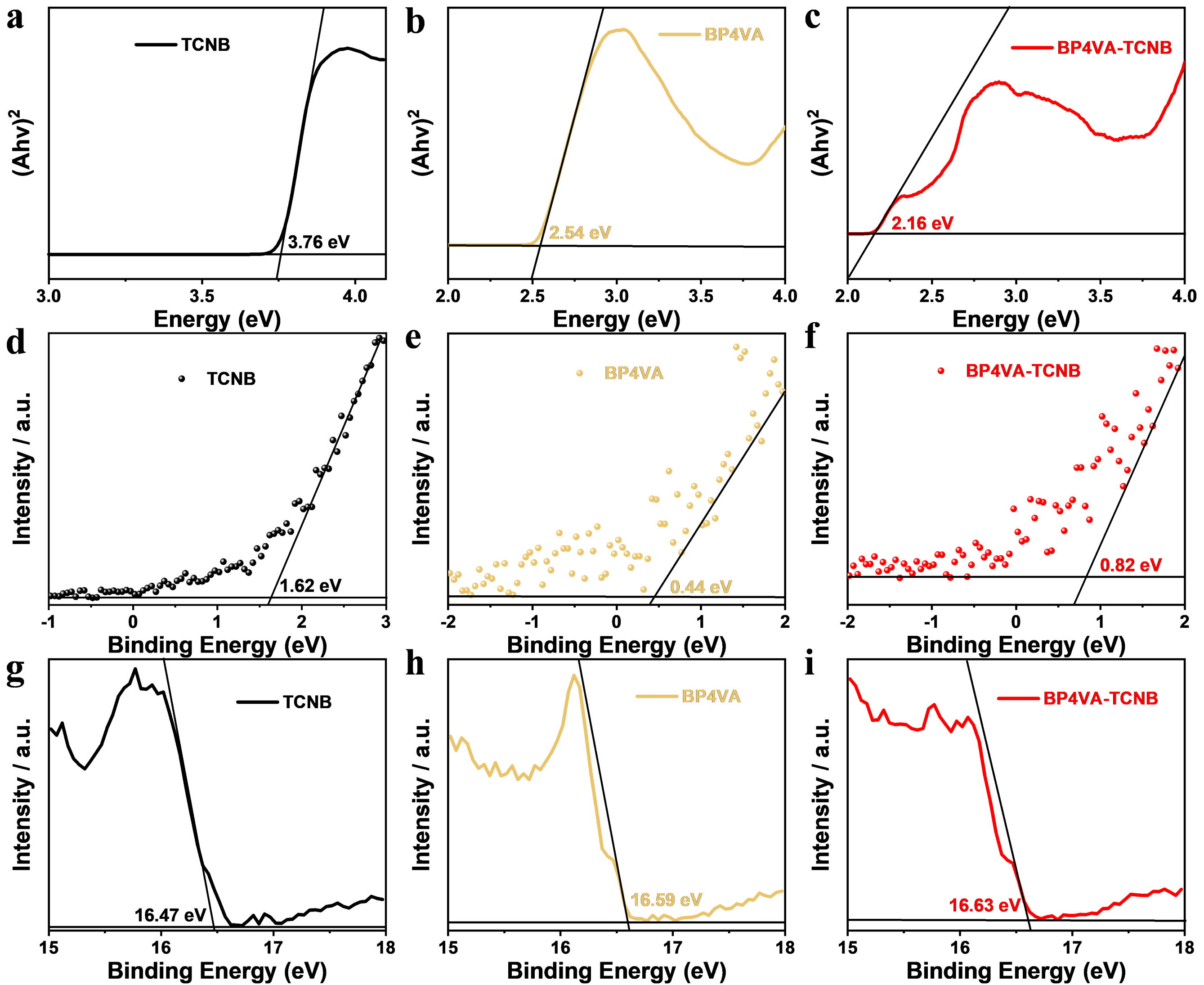


**Figure S6.** UPS energy spectra of monomers. Tauc plots for the band gap energy (*E*_g_) of (a) TCNB, (b) BP4VA, (c) BP4VA-TCNB. *E*_onset_ of (d) TCNB, (e) BP4VA, (f) BP4VA-TCNB. *E*_cutoff_ of (g) TCNB, (h) BP4VA, (i) BP4VA-TCNB.

# 11. X-ray photoelectron spectroscopy (XPS)

X-ray photoelectron spectroscopy and ultraviolet photoelectron spectroscopy analyses were performed on an ESCALAB-Xi system at Analysis and Testing Center, Tianjin University. For XPS characterization, measurements concentrated on both survey scans and high-resolution spectral acquisition within the nitrogen 1s binding energy range (396-404 eV) using X-ray excitation. Single-component materials were commercially sourced as powders from chemical suppliers, whereas co-crystalline specimens required mechanical processing of laboratory-grown single crystals before spectral interrogation.

# 12. Two photon absorption property

Cocrystal imaging via two-photon microscopy was performed using a Chameleon femtosecond laser-equipped confocal system (80 MHz repetition rate, 700-1000 nm tuning range, 140 fs pulse width) at the Technical Institute of Physics and Chemistry, Chinese Academy of Sciences. Initial sample preparation involved focal plane optimization followed by laser power quantification with a calibrated meter. Continuous laser gain modulation maintained constant output power across wavelength variations. Dual-photon confocal imaging commenced following these parameter calibrations.


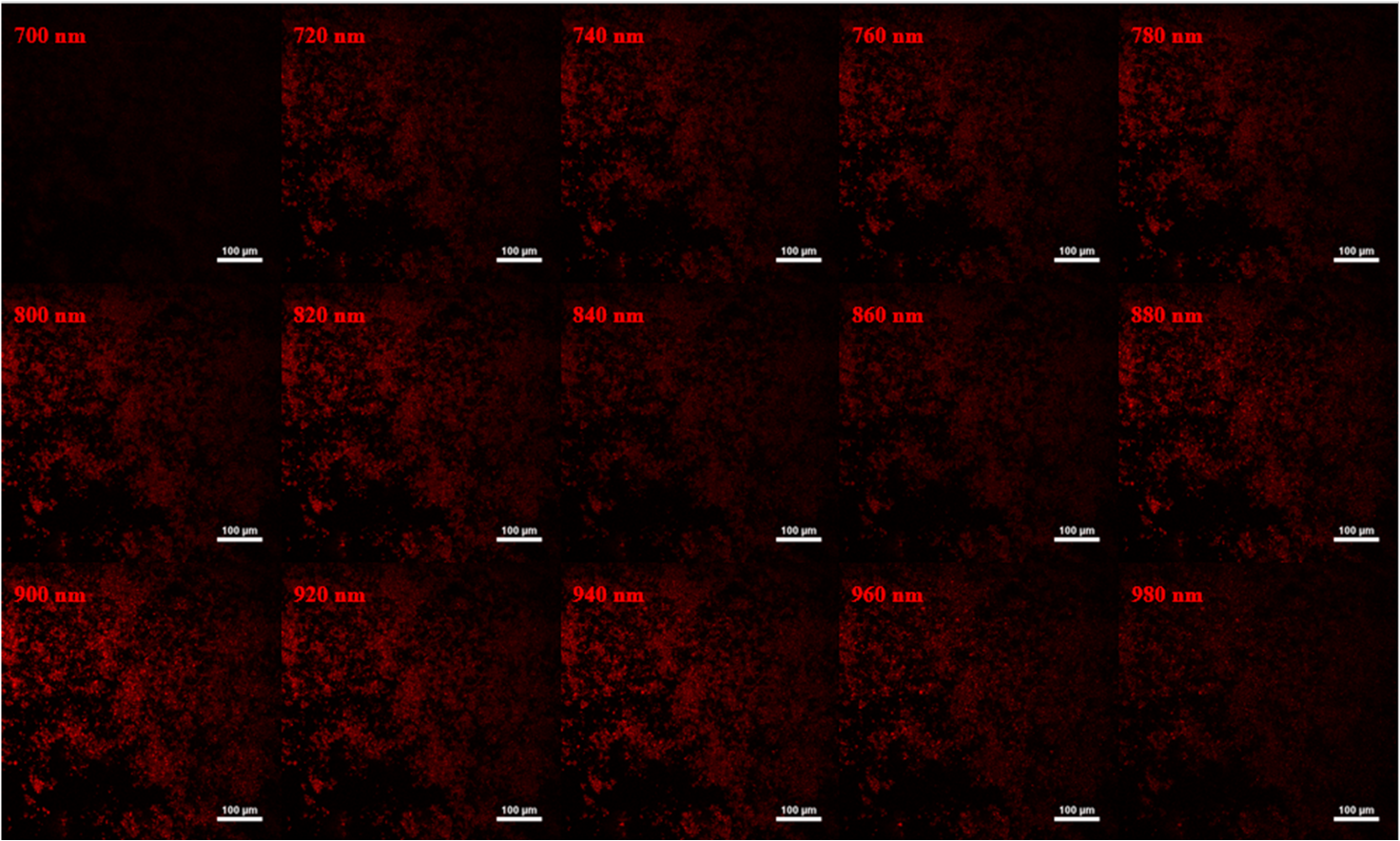


**Figure S7.** Two-photon confocal microscopy images of BP4TC cocrystal powder in the range of 700-980 nm. The optimal two-photon excitation wavelength is around 900 nm.


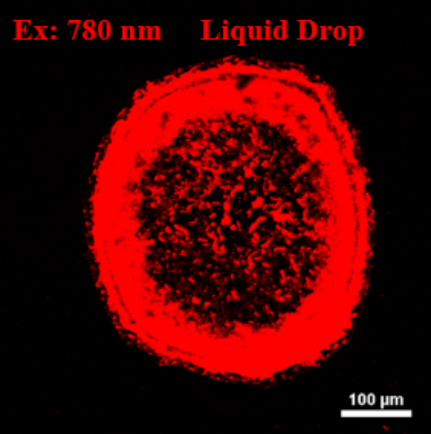


**Figure S8.** Two-photon confocal images of organic cocrystal nanoparticles in liquid state.

# **13. Two-photon excited emission spectra**

Up-converted photoluminescence and two-photon excitation spectra were measured using custom-integrated instrumentation comprising a Chameleon femtosecond laser (80 MHz repetition rate, 700-1000 nm tuning range, 140 fs pulse width) coupled to an Omni-lambda 300 spectral acquisition module at the Institute of Physics and Chemistry Technology, Chinese Academy of Sciences.

# 14. Fs transient absorption (TA) spectroscopy

Transient absorption measurements in the femtosecond regime were conducted using an instrument comprising a regenerative-amplified Ti:sapphire laser (Coherent Inc.) coupled to a Helios pump-probe detection system (Ultrafast Systems) at the Technical Institute of Physics and Chemistry, Chinese Academy of Sciences. The Ti:sapphire source (Legend Elite-1K-HE model) operated at 800 nm center wavelength with 25 fs pulse duration, 4 mJ pulse energy, and 1 kHz repetition frequency. The fundamental output was split into two optical paths: the primary beam underwent frequency conversion via an optical parametric amplifier to generate pump pulses, while a minor fraction was directed into the transient absorption spectrometer to produce probe continuum.

**Sample preparation:** To characterize the intrinsic excited-state properties of the material, pristine cocrystal specimens grown via solvent evaporation were subjected to direct analysis.


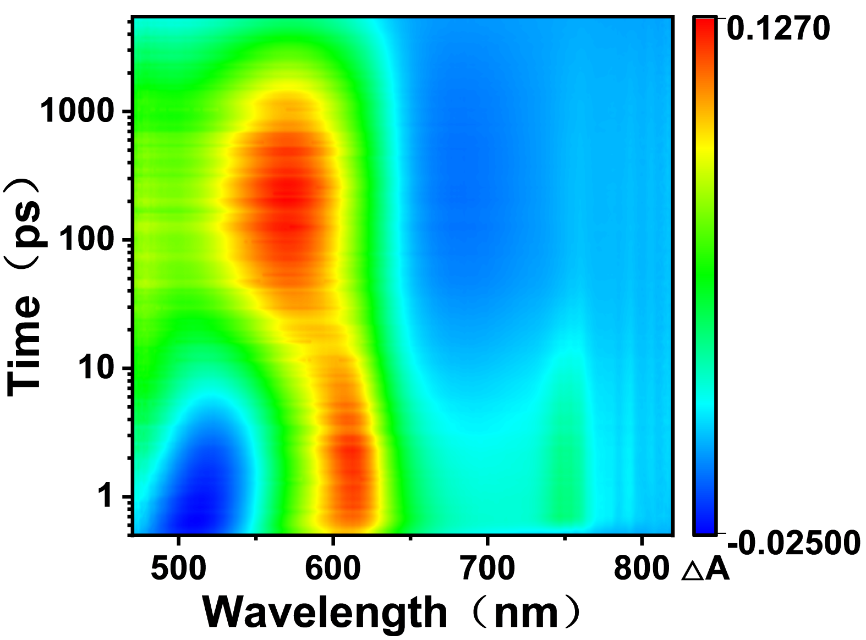


**Figure S9.** Transient absorption spectrum of BP4VA in dichloromethane (0.1 mmol/mL) with an excitation light power of 50 μW.

# 15. Z-Scan Nonlinear Optical Absorption Measurements

To systematically investigate the nonlinear optical (NLO) properties of BP4TC-NPs, we employed a home-built open-apertured (OA) Z-scan technique. The experimental setup utilized a tunable laser generated from an optical parametric amplifier (Orpheus, Light Conversion) pumped by a 1030-nm Yb:kGW amplified laser source (Pharos, Light Conversion), with a pulse width of ~200 fs and repetition rate of 750 kHz. The temporal pulse profile was characterized using an autocorrelator, confirming Gaussian pulse approximation. Samples were translated along the beam propagation axis (z-direction) using a motorized stage (LTS150/M, Thorlabs) with a 150 mm travel range and <5 μm positioning repeatability to capture complete NLO response profiles. The transmission and reference signals are collected through silicon photodiodes (PDA100A2, Thorlabs). System calibration is confirmed using carbon disulfide (CS_2_) samples.

**Sample preparation:** All samples were confirmed to satisfy the thin-sample approximation (L << z0) with 1-mm-quartz-cuvette, ensuring minimal diffraction artifacts. Experiments were conducted under thermal-stabilized conditions (25 ± 0.5°C) on a vibration-isolated optical table.

**Data fitting:**

Nonlinear optical absorption coefficients were extracted through numerical fitting to the propagation equation^[8]^,

$$\frac{dI(z)}{dz}=-\alpha\left( I \right)I$$

where *I* denotes the incident intensity, *z* represents the propagation distance within the sample. The absorption coefficient
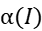
 can be described as:

$$\alpha\left( I \right)=\alpha_{0}+\beta I$$

where α_0_ and *β* are the linear and non-linear absorption coefficients, respectively. When two-photon absorption (TPA) dominates, *β* stands for TPA coefficient that can be obtained through the OA Z-scan data.

For saturation absorption, the absorption coefficient can be written as,

$$\alpha\left( I \right)=\frac{\alpha_{0}}{1+I/I_{sat}}$$

where *I*_sat_ is the saturation intensity, which is defined as the intensity value when the absorption coefficient decreases to half that of the linear absorption coefficient. When saturation absorption and TPA coexist, the absorption coefficient can be written as,

$$\alpha\left( I \right)=\alpha_{0}+\beta I+\frac{\alpha_{0}}{1+I/I_{sat}}$$

In the formula for calculating the two-photon absorption cross section, δ_2PA_ is derived from β using the following expression^[9]^, in which N_A_ denotes Avogadro’s number, c is the sample concentration, and *h*ν corresponds to the photon energy of the excitation light.

$$\delta_{2PA}=\frac{h\nu\beta}{N_{A}c}$$

To enable a direct comparison, the TPA cross-section of the BP4VA monomer was obtained experimentally (see table). The results show that the cocrystal has a higher TPA cross-section than the monomer, with a more noticeable enhancement at shorter wavelengths.

| Excitation wavelength (nm) | Experimental TPA cross-section δ_2PA_ (GM) of BP4VA NPs | Experimental TPA cross-section δ_2PA_ (GM) of BP4TC NPs |
| --- | --- | --- |
| 700 | 72.41 | 272 |
| 800 | 54.45 | 85 |
| 900 | 42.95 | 56 |
| 1000 | 29.71 | 35 |

**Table S2**. Experimental TPA cross-section δ_2PA_ (GM) of BP4VA NPs (The concentration of BP4VA is 6.580×10^-4^ mol/L)

# 16. Two photon excited fluorescence imaging of A549

The single-photon cell imaging and two photon excited fluorescence imaging were obtained using the Nikon-ARsiMP-LSM-Kit-Legend Elite-USX dual-photon confocal microscope system (Technical Institute of Physics and Chemistry, Chinese Academy of Sciences). First, the 10 uL nanoscale cocrystals samples were introduced into 990 uL fresh culture medium and thoroughly mixed them. Then, we used a pipette to separate human lung cancer cell line A549 (**RRIDs: CVCL_0023**) from the original culture medium. Subsequently, we added the fresh culture medium containing the cocrystals into the A549 cells and incubated for 10 minutes. After 10 minutes, the culture medium was removed and replaced with 10 mM phosphate buffer solution (PBS) for two photon excited fluorescence imaging. A549 cell line was purchased from iCell (Shanghai) Bioscience Inc.

**STR genotyping report of A549 cell line:** The DNA profile of this cell sample shows a complete match with an existing cell line in the reference database. According to the DSMZ database, the cell line name is A-549, corresponding to cell number 107. No additional alleles were detected in this cell line. After completing the STR test, all subsequent experiments were performed using cells directly derived from the same batch, ensuring consistent and reliable passage history and cell quality.

Genotyping results:

| Loci | Submitted Sample STR Profile | | | Reference Cell Bank STR Profile | | |
| --- | --- | --- | --- | --- | --- | --- |
|  | Sample Name：A-549 | | | Cell Bank Name：A-549 | | |
|  | Allele1 | Allele2 | Allele3 | Allele1 | Allele2 | Allele3 |
| D5S818 | 11 | 11 |  | 11 | 11 |  |
| D13S317 | 11 | 11 |  | 11 | 11 |  |
| D7S820 | 8 | 11 |  | 8 | 11 |  |
| D16S539 | 11 | 12 |  | 11 | 12 |  |
| VWA | 14 | 14 |  | 14 | 14 |  |
| TH01 | 8 | 9.3 |  | 8 | 9.3 |  |
| AMEL | X | Y |  | X | Y |  |
| TPOX | 8 | 11 |  | 8 | 11 |  |
| CSF1PO | 10 | 12 |  | 10 | 12 |  |
| D12S391 | 18 | 18 |  |  |  |  |
| FGA | 23 | 23 |  |  |  |  |
| D2S1338 | 24 | 24 |  |  |  |  |
| D21S11 | 29 | 29 |  |  |  |  |
| D18S51 | 14 | 17 |  |  |  |  |
| D8S1179 | 13 | 14 |  |  |  |  |
| D3S1358 | 16 | 16 |  |  |  |  |
| D6S1043 | 11 | 13 |  |  |  |  |
| PENTAE | 7 | 11 |  |  |  |  |
| D19S433 | 13 | 13 |  |  |  |  |
| PENTAD | 9 | 9 |  |  |  |  |
| D1S1656 | 17 | 18.3 |  |  |  |  |

**Table S3**. Genotyping results of A-549.

Genotyping Electropherogram:


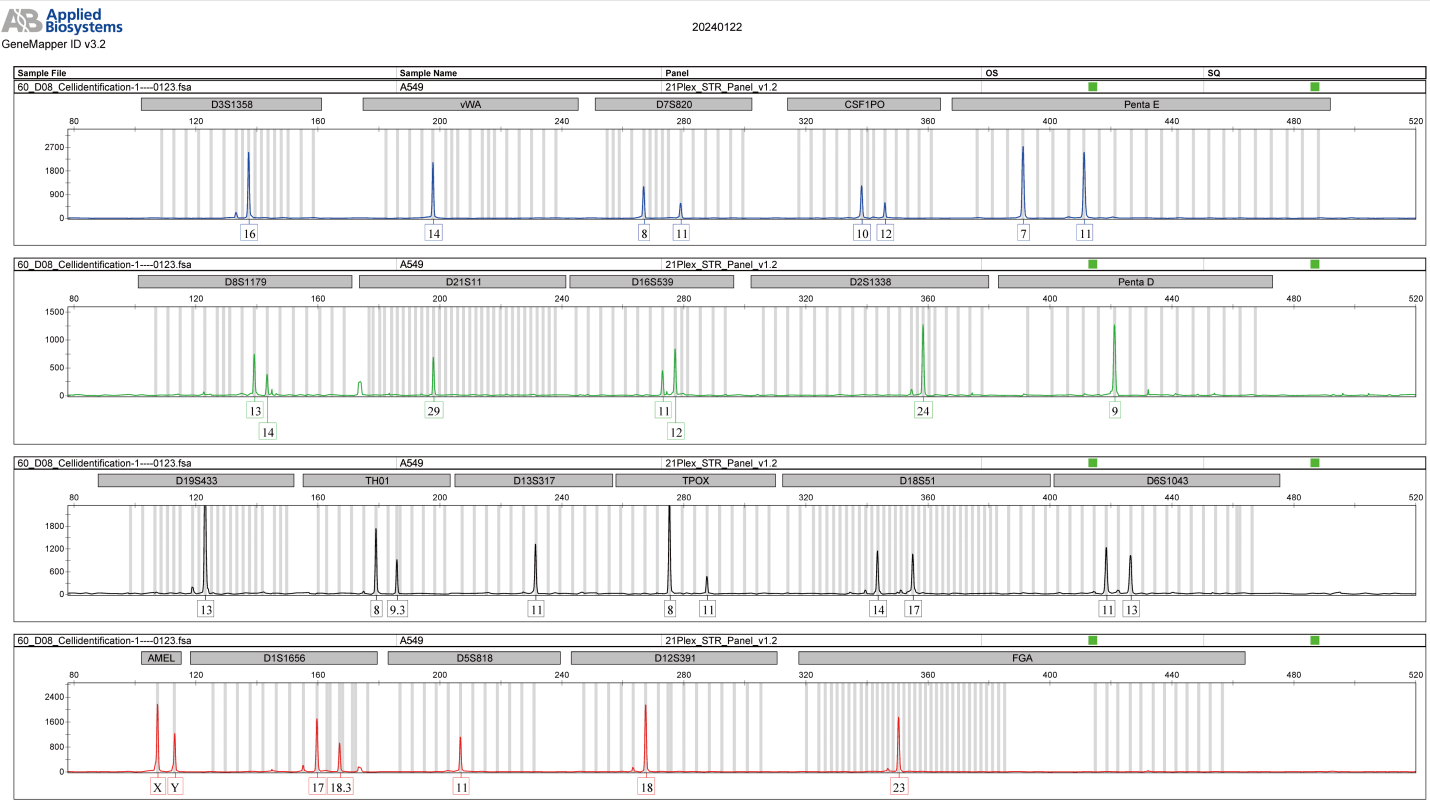


**Figure S10**.Genotyping Electropherogram of A-549.

**Cytotoxicity tests on A549 cells:** A549 cells were cultured in a 5% CO₂, 37°C incubator. Liquid samples (5 mg/mL) were sterilized by UV light for 30 minutes. The cells were divided into Control, Sample, and 40% pure water groups. In the Control group, complete culture medium was added; in the Sample group, various concentrations of sample working solutions were added; and in the 40% pure water group, solutions containing 40% pure water were added. Cells in the logarithmic growth phase were counted, adjusted to 4 × 10³ cells per well, and seeded into a 96-well plate, cultured overnight. After 24 hours of treatment, the medium was removed, and wells were washed with PBS. Then, 120 μL of medium containing 10% CCK-8 was added, and the cells were incubated for 2 hours. Absorbance at 450 nm was measured using a microplate reader.

**Cell Live/dead staining experiment:** A549 cells were cultured in a 5% CO₂, 37°C incubator. Liquid samples (5 mg/mL) were sterilized by UV light for 30 minutes and diluted with complete medium. Cells were divided into Control and Sample groups, with the Control group receiving complete medium and the Sample group receiving 10 μg/mL of the sample solution. A549 cells in the logarithmic growth phase were seeded at 6 × 10⁴ cells per well in confocal dishes and cultured overnight. After 24 hours, cells were washed with PBS, stained with a dye solution (Calcein-AM and PI), incubated for 15 minutes, and washed again. Staining results were observed using a laser confocal microscope.


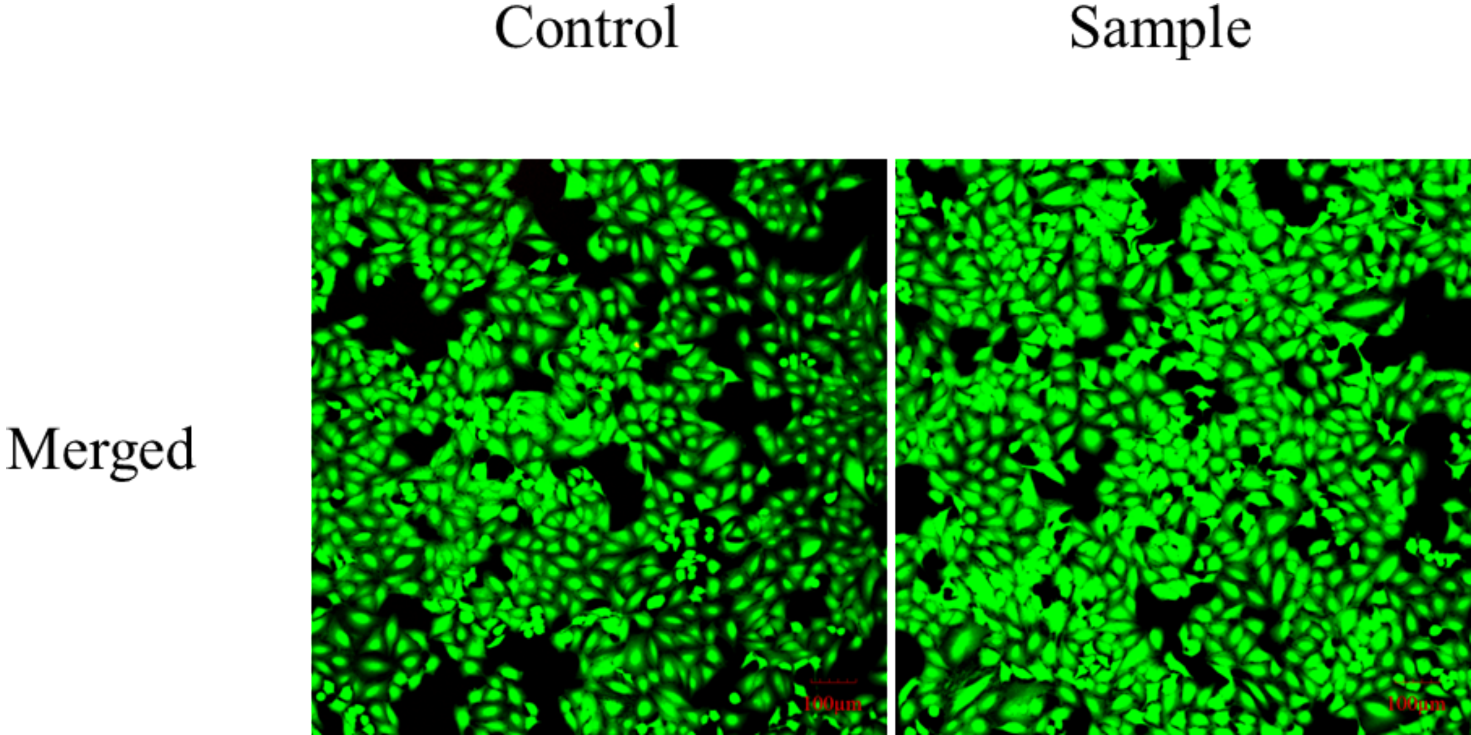


**Figure S11**. The cell viability of A549 cells after co-culturing with the sample for 24 hours (100X).

# 17. Stability assessment of BP4TC-NPs in physiological conditions

The particle size measurements were performed on a Zetasizer Nano ZS90 instrument at Analysis and Testing Center of Tianjin University, utilizing the dynamic light scattering (DLS) technique. Nanoparticles were dispersed in PBS buffer for testing.

| Time (h) | Z-Average (d. nm) | PDI |
| --- | --- | --- |
| 0 | 46.37 | 0.237 |
| 12 | 44.23 | 0.236 |
| 24 | 42.2 | 0.232 |
| 36 | 43.59 | 0.217 |
| 48 | 45.02 | 0.236 |

**Table S4.** Size Change of BP4TC-NPs in PBS from 0 to 48 h.

# 18. First-principles calculations

| Cocrystal and their components | Calculated Bandgap/eV |
| --- | --- |
| BP4VA-TCNB | 1.04 |
| BP4VA_*P*2_1_/*c* | 1.71 |
| TCNB | 3.00 |

**Table S5.** Calculated bandgap of cocrystal BP4VA-TCNB and their monomers.

| Cocrystal and their components | Δμ |
| --- | --- |
| BP4TC | 47.06 |
| 1D-BTC | 12.40 |
| 2D-BTC | 23.99 |
| 1,2-BTC | 28.81 |

**Table S6.** Calculated difference in dipole moments between the ground and excited states (Δμ) of BP4VA-TCNB compared with other cocrystals^[7]^.

# References

[1] Wen, M. J., Jackson, M. T. & Garner, C. M. A quantitative study of vapor diffusions for crystallizations: rates and solvent parameter changes. *Dalton Trans.* **2019**,48, 11575.

[2] Cui, J., Zhang, F., Yan, D., Han, T., Wang, L., Wang, D. & Tang, B. Z. “Trojan Horse” Phototheranostics: Fine-Engineering NIR-II AIEgen Camouflaged by Cancer Cell Membrane for Homologous-Targeting Multimodal Imaging-Guided Phototherapy. *Advanced Materials* **2023**,35, 2302639.

[3] Tian, S., Bai, H., Li, S., Xiao, Y., Cui, X., Li, X., Tan, J., Huang, Z., Shen, D., Liu, W., Wang, P., Tang, B. Z. & Lee, C.-S. Water-Soluble Organic Nanoparticles with Programable Intermolecular Charge Transfer for NIR-II Photothermal Anti-Bacterial Therapy. *Angewandte Chemie International Edition* **2021**,60, 11758.

[4] Lin, R., Liu, J., Xu, W., Liu, Z., He, X., Zheng, C., Kang, M., Li, X., Zhang, Z., Feng, H.-T., Lam, J. W. Y., Wang, D., Chen, M. & Tang, B. Z. Type I Photosensitization with Strong Hydroxyl Radical Generation in NIR Dye Boosted by Vigorous Intramolecular Motions for Synergistic Therapy. *Advanced Materials* **2023**,35, 2303212.

[5] Ou, C., Na, W., Ge, W., Huang, H., Gao, F., Zhong, L., Zhao, Y. & Dong, X. Biodegradable Charge-Transfer Complexes for Glutathione Depletion Induced Ferroptosis and NIR-II Photoacoustic Imaging Guided Cancer Photothermal Therapy. *Angewandte Chemie International Edition* **2021**,60, 8157.

[6] Jiang, Y., Upputuri, P. K., Xie, C., Lyu, Y., Zhang, L., Xiong, Q., Pramanik, M. & Pu, K. Broadband Absorbing Semiconducting Polymer Nanoparticles for Photoacoustic Imaging in Second Near-Infrared Window. *Nano Lett.* **2017**,17, 4964.

[7] Hao, L., Liu, F., Wang, X., Kang, L., Wang, Y., Wang, L., Lin, Z. & Zhu, W. Crystallography, Charge Transfer, and Two‐Photon Absorption Relations in Molecular Cocrystals for Two‐Photon Excited Fluorescence Imaging. *Small* **2024**,20, 2308470.

[8] Sheik-Bahae, M., Said, A. A., Wei, T.-H., Hagan, D. J. & Van Stryland, E. W. Sensitive measurement of optical nonlinearities using a single beam. *IEEE Journal of Quantum Electronics* **1990**,26, 760.

[9] Feng, W., Liu, K., Zang, J., Wang, G., Miao, R., Ding, L., Liu, T., Kong, J. & Fang, Y. Flexible and transparent oligothiophene‑o‑carborane-containing hybrid films for oonlinear Optical Limiting Based on Efficient Two- Photon Absorption. *ACS Appl. Mater. Interfaces* **2021**,13, 28985.
